# Raman Microspectroscopy to Trace the Incorporation of Deuterium from Labeled (Micro)Plastics into Microbial Cells

**DOI:** 10.1021/acs.analchem.4c05827

**Published:** 2025-02-10

**Authors:** Kara Müller, Martin Elsner, Anna E. Leung, Hanna Wacklin-Knecht, Jürgen Allgaier, Maria Heiling, Natalia P. Ivleva

**Affiliations:** † Chair of Analytical Chemistry and Water Chemistry, School of Natural Sciences, Technical University of Munich, Lichtenbergstr. 4, Garching 85748, Germany; ‡ Scientific Activities Division, European Spallation Source ERIC, Lund 221 00, Sweden; § Division of Physical Chemistry, Department of Chemistry, Lund University, P.O. Box 124, Lund SE-22100, Sweden; ∥ Jülich Centre for Neutron Science (JCNS-1), Forschungszentrum Jülich GmbH, Jülich 52428, Germany; ⊥ Soil and Water Management and Crop Nutrition Laboratory, Joint FAO/IAEA Centre of Nuclear Techniques in Food and Agriculture, International Atomic Energy Agency (IAEA), Vienna 1400, Austria

## Abstract

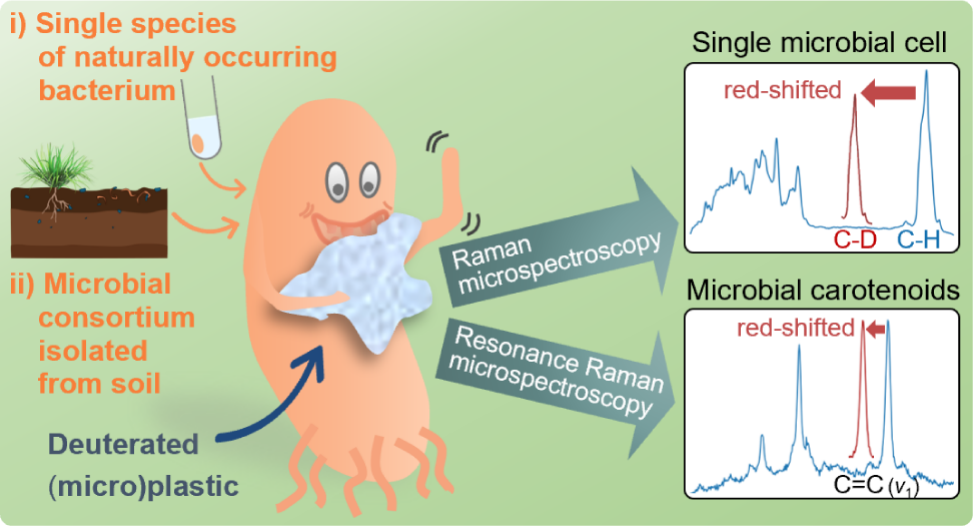

The ubiquitous use of plastics demands thoughtfulness about their fate in the environment. Biodegradability is, therefore, a prerequisite for the future use of plastics in many applications, including agriculture. Here, we bring forward stable isotope (resonance) Raman microspectroscopy at the single-cell level to broaden the mechanistic understanding of microbial degradation of (micro)plastics in natural systems. We selected perdeuterated d-polylactic acid (dPLA) as model plastic, synthesized from d-lactic acid-d_4_, via enantioselective, biocatalytic reduction of pyruvate-d_3_. With dPLA in hand, we traced the deuterium label during incubation experiments into microbial biomass using C–D vibrations (appear in the Raman-silent region of undeuterated biomass, 2050–2300 cm^–1^). The 2068 cm^–1^ C–D band was indicative of strongly deuterated lipids enabling the detection of metabolic differences during incubation with dPLA (i.e., stronger lipid and weaker protein deuteration) compared to glucose-d_12_ and D_2_O as alternative D sources. Single-cell analysis was the key to detecting phenotypic heterogeneity and classifying cells of the naturally occurring bacterium *Sphingomonas koreensis* in two clusters: one showed a significantly stronger deuteration level than the *Escherichia coli* control, whereas the other was nonlabeled. The deuterium label could even be detected in the strong resonance Raman signal of carotenoids, highlighting the potential for high throughput technologies like imaging and cell sorting. To further demonstrate the transferability to environmental samples, the experiment was repeated with soil bacteria isolates, and deuterium uptake from dPLA into microbial biomass was observed after 2 weeks.

## Introduction

Because of the ease with which their properties can be tailored, polymers are ubiquitous worldwide. For instance, agricultural plastics increase crop yields and quality and reduce water and pesticide demand.^1−3^ However, many products cannot be completely retrieved and accumulate in agricultural soils.^4−8^ Their presence leads to physical, chemical, and biological impacts on organisms and soil health.^1,9−12^ Since the agricultural use of plastics is unlikely to stop soon, they cannot be prevented from ending up in soil environments. Completely biodegradable polymers are, therefore, a prerequisite for sustainable future applications.

To this end, the biodegradation process must be systematically studied under environmentally relevant conditions to reliably classify biodegradable polymers and to design new ones. First, the polymers are fragmented^13,14^ and must be then broken down into smaller oligo- and monomers for microbial utilization.^3,15,16^ The initial depolymerization can either be biotic (extracellular enzymatic depolymerization) or abiotic (hydrolysis).^3,17,18^ In both cases, the subsequent microbial conversion into CO_2_, water, and biomass can be monitored to estimate the final fate of the plastic in the investigated environment. Quantitative CO_2_ measurements have been performed compared to controls,^19,20^ where little attention was paid, however, to whether other processes might be triggered due to bacteria adaptation that would also produce CO_2_, leading to wrong positive results. ^14^C- or ^13^C-labels,^15,21,22^ are more adequate to avoid such bias. However, not all polymer carbon is transferred into CO_2_. A fraction that depends on species, substrates, and environmental conditions is channeled into biomass. This anabolic contribution is essential for a complete picture of plastic biodegradation. The first mass balance reported by Nelson et al. included ^13^CO_2_ measurements, extracting the residual polymer with subsequent quantitative ^1^H NMR, and estimating the biomass formation.^21^ However, this approach entails considerable effort, is associated with significant uncertainties in the case of small degrees of degradation, and does not deliver information about responsible species and their metabolism. This illustrates the value gained if the biomass is measured directly.

Isotopic labels can trace element fluxes from a polymer into microbial biomass, thereby labeling responsible species. Sander called for new isotope methods to analyze the biodegradability of (micro)plastics in soil environments.^3^ Zumstein et al. used ^13^C-labels in combination with nanoscale secondary ion mass spectrometry (NanoSIMS) to trace carbon from polymer into fungal biomass.^15^ NanoSIMS can directly measure distributions of several elements in parallel, achieving a very high sensitivity and lateral resolution down to 50 nm (below single-cell resolution). However, the method is highly time- and cost-intensive, restricting the number of measurements and impeding statistical representativity.^23^ Additionally, the sample’s topography can influence measured isotope ratios,^24^ and there might be isotope carryover from the underlying substrate.^15^ Goudriaan et al. traced ^13^C from labeled polyethylene into ^13^CO_2_ and microbial lipids.^22^ The uptake into microbial lipids was then used to estimate ^13^C assimilation in the entire microbial biomass, although uncertainties remain since no information was gained about other biomolecules. Consequently, alternative analytical approaches are warranted to detect isotope labels from the polymer within the biomass.

**Figure 1:**
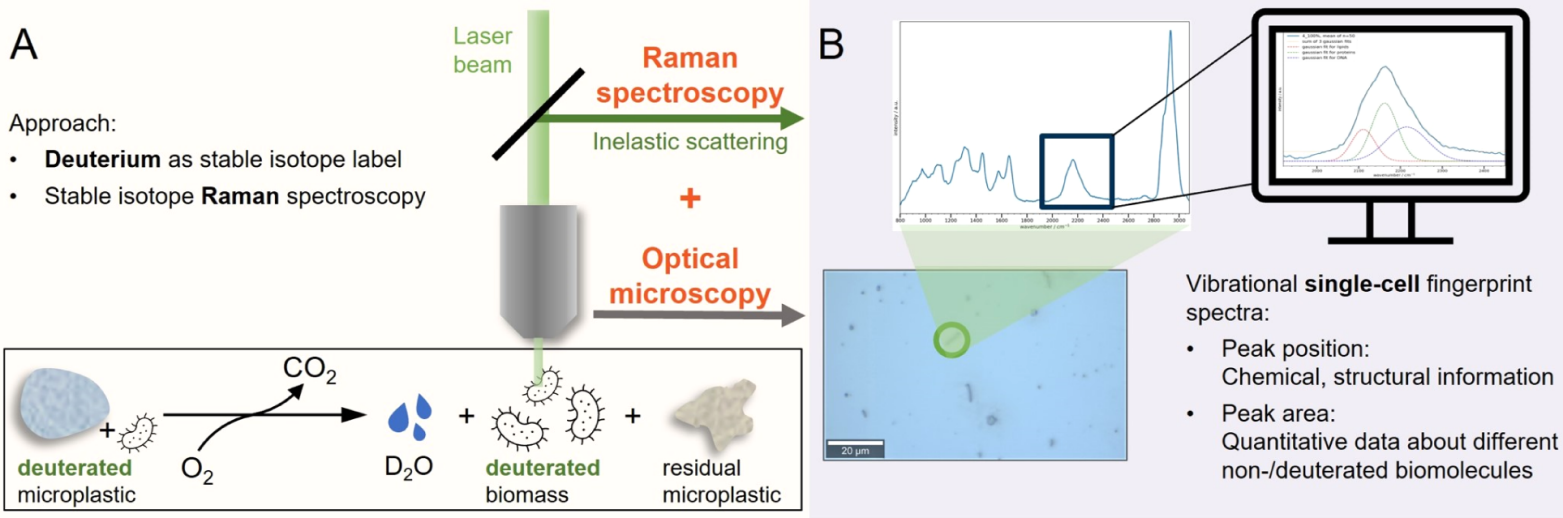
(A) Stable isotope Raman microspectroscopy is used to trace deuterium from labeled (micro)plastic into biomass with the aim of following this process during biodegradation. (B) This novel approach provides vibrational single-cell spectra in combination with their optical images. Peak fitting gives the peak position as a qualitative and the peak area as a quantitative measure.

Spectroscopic methods have been demonstrated to trace stable isotope-labeled element fluxes. Specifically, coupling an optical microscope to a Raman spectrometer gives vibrational fingerprint spectra with a lateral resolution down to 1 μm (Figure 1). Due to their mass difference, heavier isotopes show red-shifted Raman bands (lower wavenumbers), which indicate the isotope label when present in polymers or biomass. For example, deuteration leads to a decisive shift of the C–H Raman band into the Raman-silent region of nondeuterated microbial biomass. After simple sample preparation, single cells/particles can be measured in high quantities to account for statistical representativity. Despite its low quantum efficiency and sensitivity, stable isotope Raman microspectroscopy (SIRM) can be applied to environmental samples. Since water is a very weak Raman scatterer, it does not interfere with the analysis, so direct measurements of aquatic samples are possible.^25−29^ Moreover, SIRM has been demonstrated for soil bacteria after isolating cells from the matrix.^30−35^

We have not found any SIRM studies about the microbial uptake of labeled plastics. Nevertheless, SIRM was previously applied for the degradation analysis of other labeled substrates and environmental pollutants and validated with mass-based methods.^23,24,36,37^ Specific metabolic pathways and degradation of environmental pollutants are commonly studied with ^13^C-labeled substrates (i.e., glucose metabolism,^30,38−41^ naphthalene or other polycyclic aromatic hydrocarbons,^33,41,42^ toluene^34^ or phenol^36^ degradation, and CO_2_ fixation.^28,43,44^ Moreover, ^13^C-labels have been used to monitor microbial growth rates,^45^ microbial proteome dynamics,^40^ and carbon flow in a food chain.^46^ Compared to specific ^13^C-labeled substrates, heavy water can serve as a general indicator of metabolic activity as it is incorporated into biomass during lipid and protein biosynthesis.^37,47,48^ For example, this approach enables the analysis of metabolic activity in response to antibiotics for studying antimicrobial resistance^25,35,49−51^ or in response to environmental changes such as droughts.^52^ While ^13^C-labels may appear to be the more intuitive choice for studying specific metabolisms as they get less diluted during biosynthesis pathways and there is no isotope exchange, D-labels can also be used for this purpose. Therefore, nonlabeled carbon substrates can be applied with D_2_O,^53,54^ or deuterated substrates can be used directly (e.g., glucose and naphthalene,^30,54−56^ or fatty acids.^57,58^ Choosing D-labels results in a more pronounced isotope shift compared to ^13^C-labels, preventing overlaps with the original undeuterated C–H vibrations. This allows to get more information from the band shape (position and area) with the potential to facilitate applications like Raman cell sorting^28,47,59−62^ to separate plastic-degrading bacteria from an environmental microbial consortium, allowing for subsequent species identification or further analytics. Furthermore, given the larger natural abundance of deuterium than ^13^C, coupled with the generally easier enrichment of organic molecules with this label, deuterated substrates frequently exhibit lower cost and sometimes enhanced availability. For fully labeled pyruvate, the precursor for polylactic acid (PLA), the polymer used in this study, the perdeuterated molecule is about a third of the price of the ^13^C-labeled one.^63^

We synthesized perdeuterated polylactic acid (dPLA) to analyze its incorporation into microbial biomass in incubation experiments. PLA, the bioplastic with the greatest share of global production in 2022,^64^ is used as mulching films, twines, and nets in agriculture^1^ and, therefore, has a significant environmental impact. The naturally occurring bacterium *Sphingomonas koreensis* (*S. koreensis*) was isolated from a PLA suspension^38^ and applied as a first model species. *Sphingomonas* have been previously shown to enrich in PLA/PBAT mulch contaminated soils^65^ and in water filter systems made of plastics^66,67^ due to its biofilm-forming ability on plastic surfaces^66,68^ and versatile metabolism.^66,67^*S. koreensis* produces carotenoids that have a chromophore system and show a resonance Raman effect, which is orders of magnitude more sensitive than spontaneous Raman.^38,43,69,70^ Therefore, we also explored resonance Raman spectroscopy of deuterated microbial carotenoids.

In this study, we aim to provide a complementary method for directly demonstrating that microbes utilize building blocks from the polymer to build up biomass as part of plastic biodegradation. To this end, we trace deuterium from the labeled polymer into individual bacterial cells with Raman microspectroscopy (Figure 1). This method was developed for a naturally occurring model bacterium and later applied to a soil-derived bacterial consortium. Unlike nanoSIMS our spectroscopic approach offers chemical information based on vibrational bands, with the potential to enable insight into the types of biomolecules that take up the deuterium in bacterial metabolism. Most importantly, this methodology allows for the measurement of a statistically representative number of single cells to take into account phenotypic heterogeneity.

## Experimental Section

### Deuterated Polylactic Acid (dPLA)

The synthesis of d-lactic acid-d_4_, d,d-lactide-d_8_, and the subsequent polymerization to dPLA is in preparation for publication elsewhere.^71^ Briefly, d-lactic acid-d_4_ was synthesized from sodium pyruvate-d_3_ using d-lactate dehydrogenase from *Lactobacillus leichmanii*, NAD^+^, sodium formate-d_1_ and formate dehydrogenase from *Candida boidinii*. d-d-lactide-d_8_ was synthesized according to a literature procedure^72^ from d-lactic acid-d_4_ in the presence of zeolite beta (Si/Al = 12.5) as a shape-selective catalyst. This process allows lactide synthesis in high yields and avoids racemization reactions. Anionic d-d-lactide-d_8_ polymerization provided perdeuterated d-polylactic acid (dPLA). Diethylzinc and benzyl alcohol were used as initiator system and the polymerization reactions were carried out in dry tetrahydrofuran using standard Schlenk techniques. Finally, the polymer was purified and isolated by precipitation in hexane/isopropanol (90 vol %/10 vol %) and vacuum drying. The polymer was used without any further processing steps for biodegradation experiments.

The number-average molecular weight (*M*_n_) of the dPLA sample is *M*_n_ = 3900 g/mol with narrow molecular weight distribution (*M*_w_/*M*_n_ = 1.16, with *M*_w_ being the weight-average molecular weight), as measured by size exclusion chromatography (SEC) using an Agilent 1260 Infinity SEC instrument equipped with a Wyatt DAWN Heleos II light scattering (LS) detector, an Optilab T-rex differential refractive index (RI) detector and with three PolyPore columns at 50 °C (see Figure S16 for SEC RI-trace of dPLA). The dPLA used in this study has a relatively low molecular weight compared to commercial materials (typically 1,000 to several 10 000 g/mol). However, using highly pure d,d-lactide-d_8_ is anticipated to result in a high degree of crystallinity, as evidenced by a high melting point (160 °C) observed in a similar dPLA sample produced under identical conditions. High crystallinity enhances the polymer’s mechanical strength,^73^ which is well-suited for agricultural applications.

Secondary electron microscopy (SEM) was used to estimate the dPLA particle size distribution (after particle deagglomeration by short (45 s) treatment in an ultrasonic bath) (see Figures S13–S15), and particles with a maximal diameter of 60 nm to 28 μm were found with an irregular shape.

### Incubation Experiments with Monospecies

Unless otherwise stated, all chemicals and materials were purchased from Carl Roth (Karlsruhe, Germany) and Sigma-Aldrich (Steinheim, Germany). All materials were autoclaved prior to incubation experiments at 120 °C. Before biodegradation experiments, *Sphingomonas koreensis* and *Escherichia coli* (used as a control for an abiotic dPLA hydrolysis since the strain is not expected to enzymatically degrade plastics) were grown separately in an optimized minimal M9 medium (MM, see Table S3 for medium composition)^38^ supplemented with 2.5 g/L sodium L-lactate for 13 days. For the biodegradation experiments, the initial cells were washed three times in MM without an additional carbon source. They were then incubated in duplicates at room temperature of 21 °C in 10 mL organic carbon-free MM (OD600 = 0.03, pH = 7.3). The cell suspension was supplemented with 3 mg dPLA (0.3 g/L) in a 20 mL rolled-rim glass vial with aluminum foil as a lid at the beginning of the experiment. After 0, 3, and 13 weeks, 70–100 μL of cell suspension of the thoroughly mixed biological duplicates were harvested in triplicates for Raman measurements.

For the reference spectra, *S. koreensis* was incubated in the same MM, supplemented with different carbon and deuterium sources (d-glucose-d_12_ (97 atom % D, Santa Cruz Biotechnology, USA), d,d-lactide-d_8_ (dimer, which was used for dPLA synthesis), and D_2_O) in an incubator shaker at 37 °C and 120 rpm (see Table S2 for designs of varying reference experiments). The carbon sources were always added to yield a total substrate concentration of 4 g/L. All reference spectra were measured after at least 3 days of incubation so that *S. koreensis* cells reached the stationary phase.

### Incubations of Soil Microbiome with PLA

Soil samples, classified as Cambisol, were taken from Grabenegg, Austria, at 0–15 cm depth, sieved to 2 mm, and incubated with microplastic particles (sieve fraction: 250–500 μm) made of conventional PLA (Ingeo Biopolymer 2003D, NatureWorks LLC) for two years (see Table S3 for soil characteristics before incubation). During this period, the samples were kept at 22 °C, and the soil water content was periodically readjusted to 60% of the water holding capacity. Bacteria were then isolated from soil via a Nycodenz gradient separation according to Eichorst et al.^31^ (see Supporting Information for more details) and incubated in 5 mL minimal M9 medium (same composition as for *S. koreensis* incubations) with 1.4 mg dPLA, which yielded an initial OD600 of 0.2. After 2 weeks, the cells were harvested for Raman measurements.

### Single-Cell Raman Measurements

Samples were washed two times in twice the volume of Milli-Q water, and resuspended in the initial volume of Milli-Q water (see Supporting Information for washing procedure). One μL droplets were applied on Al-coated glass slides (EMF Dynasil, USA) and left to dry before the Raman measurement. Conventional biomass and resonance Raman spectra were acquired with 8 and 1 mW laser powers at the sample and acquisition times of 10 and 2 s, respectively, on an *alpha300 apyron* automated confocal Raman microscope (WiTec, Ulm, Germany). For the soil bacteria, automated Raman measurements were performed with the Witec software *ParticleScout*, which included spectral autofocus. A maximum of 15 accumulations with 0.5 s integration time and 8 mW laser power at the sample were collected per cell unless a signal-to-noise ratio of 110 was reached beforehand. During all measurements, the instrument was used with a power-adjustable frequency-doubled Nd:YAG laser (532 nm), a 300 g mm^–1^ diffraction grating, and a 100× objective (Zeiss EC Epiplan-Neofluar, numerical aperture, NA = 0.9, working distance, WD = 1 mm, Zeiss, Germany).

### Spectra Processing and Fitting Procedures

For dPLA biodegradation experiments, only biomass spectra were chosen for the analysis, and other spectra, such as plastic particles or cells showing traces of overlapping dPLA, were neglected. Single-cell Raman spectra of all replicates were combined and processed with an automated in-house Python script so that the same procedure was applied to all spectra. More details can be found in the Supporting Information. If not noted otherwise, all spectra (including the spectra of reference experiments) were preprocessed with a wavenumber correction based on a Si-wafer, rubber band background correction, and normalized to the C–D plus C–H integrals. Peak areas, bandwidth, and band positions were determined from Gaussian fits with different boundary conditions (see Supporting Information).

## Results and Discussion

### Raman Spectroscopy to Differentiate Bacteria from Microplastics

**Figure 2:**
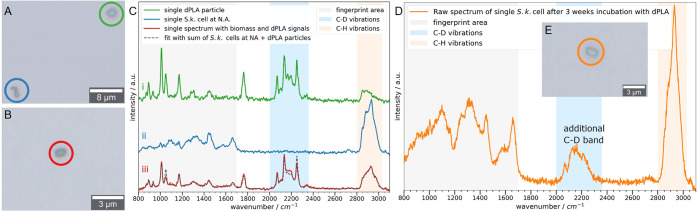
(A,B) Optical images obtained at 100× magnification of a dPLA particle (green), a *S. koreensis* cell (blue), and a cell overlapping with a dPLA particle (red) correspond to the Raman spectra of the same color in (C). The black dashed line describes a fit of the red spectrum (iii) by a weighted sum of a spectrum of *S. koreensis* cells at natural isotopic abundance with a mean spectrum of dPLA particles, according to eq 1. (D) Single-cell Raman spectrum of the cell incubated for 3 weeks with dPLA, whose optical image (100× magnification) is shown in (E).

Even when *Sphingomonas koreensis* cells and dPLA particles were well separated on the sample slide for stable isotope Raman microspectroscopy measurements (Figure 2A), it was difficult to optically differentiate them because of similar dimensions and shapes. Raman spectra, in contrast, enabled a straightforward assignment of both microscopic entities (Figure 2C (i–ii)). All 61 initial *S. koreensis* cells showed similar Raman spectra to that of the single cell depicted in Figure 2C (ii), typical for microorganisms^74−77^ with broad and overlapping Raman bands of various biomolecules. On the contrary, all 54 initially measured dPLA particles had Raman spectra like this in Figure 2C (i). The stereodefined polyester has a chemically simple repeated subunit (see Figure S1), leading to a few well-separated and sharp Raman bands. Due to their high intensities, they can even be recognized in spectra of particles that overlap with cells. Specifically, from the optical image of Figure 2B, it would not be discernible if the object is composed of different components. Here, the fingerprint region (800–1700 cm^–1^) and the C–H vibrations (2800–3500 cm^–1^) in the Raman spectrum (Figure 2C (iii)) indicate the presence of biomass (BM). Simultaneously, the C–D vibrations of dPLA (2050–2300 cm^–1^) give unequivocal evidence of the presence of a plastic particle.

Hence, the observed spectrum can be described by a fit of a weighted sum of the mean Raman spectrum of *S. koreensis* cells at natural isotopic abundance (y_BM_) and the dPLA spectrum (y_dPLA_) with the respective scaling factors f_BM_ and f_dPLA_ (eq 1). A y-offset (y_0_) is added to account for the existence of a background. Further fits are attached (Figure S2).


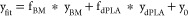
1

While SIRM cannot determine whether the overlapping dPLA is taken up by the cell or sorped onto its surface, higher resolution techniques like nanoSIMS or time-of-flight SIMS could provide this information. Nonetheless, the chemical structural information on the C–D Raman signature and the model of eq 1 were sufficient to avoid confusion between overlapping dPLA and biomass deuteration.

After 3 weeks of incubating *S. koreensis* cells with dPLA, an additional type of Raman spectrum came up (Figure 2D,E), which cannot be described by the model of eq 1.

### Incubation with dPLA Leads to a New C–D Raman Signature (2050–2300 cm^–1^) in Biomass Spectra

**Figure 3:**
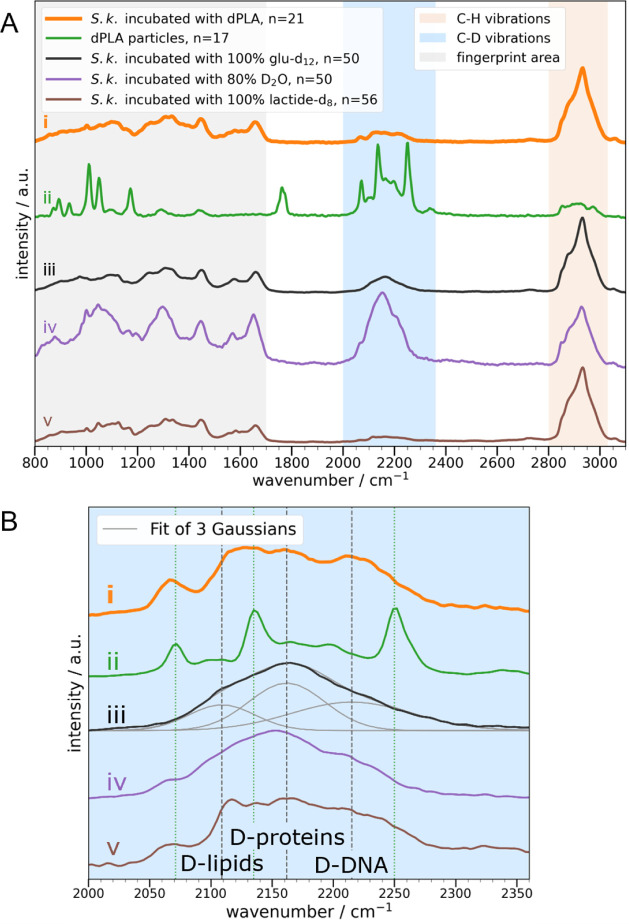
(A) Mean Raman spectrum of 21 single *S. koreensis* cells incubated with dPLA (i) in comparison to a mean spectrum of: (ii) 17 dPLA particles; and at least 50 *S. koreensis* cells labeled with (iii) glucose-d_12_; (iv) 80% D_2_O, and (v) lactide-d_8_. (B) Raman spectra from A were normalized to the C–D region (1960–2400 cm^–1^). The three Gaussian fits of the C–D region of the glucose-d_12_ reference can be assigned to the Raman bands of D-lipids, D proteins, and D-DNA.

Twenty-one cells measured after 3 weeks of dPLA incubation showed the additional C–D signature as represented in the mean spectrum (Figure 3A (i); single spectra in Figure S3). While the fingerprint area (800–1700 cm^–1^) and C–H vibrations (2800–3500 cm^–1^) indicate microbial biomass, the nature of the C–D vibrations (2050–2300 cm^–1^, Figure 3B (i)) warrants closer consideration. The latter differed significantly from the mean dPLA spectrum (Figure 3A,B (ii)). The symmetric and antisymmetric methyl C–D vibrations of dPLA^78^ at 2137 and 2250 cm^–1^ were not pronounced in the new C–D signature of the biomass spectra. Only the overtone of the dPLA asymmetric methyl C–D deformation mode at 2070 cm^–1^ coincided with a Ramanband of the new C–D signature of the *S. koreensis* spectra at 2068 cm^–1^, but their bandwidths differed significantly. For dPLA particles, it lay between 5 and 6.5 cm^–1^ even after 20 weeks of incubation with *S. koreensis* (Figures S4A–C), while it was 12 cm^–1^ for *S. koreensis* cells incubated for 3 weeks with dPLA (Figure S4D). The simple structure of the polymer, which has only one methyl group with the same stereoinformation in its monomeric unit, explains the narrow bandwidth, whereas the broad bandwidth suggests more complex entities, as typical for a diversity of biomolecules.

To test whether the entire C–D signature could originate from deuterated biomass, we traced deuterium from another carbon-bound deuterium source into microbial biomass and compared the C–D vibrations. There, the incorporated deuterium is also diluted based on water uptake during the biosynthesis of various biomolecules, which can influence the C–D signature. For this reason, the spectrum of dPLA incubation was compared to a mean spectrum of 50 *S. koreensis* cells incubated with glucose-d_12_ (Figure 3A,B (iii)). C–D signatures of deuterated biomass generally originate from the overlap of signals from a variety of biomolecules. Therefore, a sufficient number of reference spectra would be required for an accurate deconvolution, as presented by Uematsu et al., who analyzed 22 deuterated fatty acids to estimate cellular fatty acids based on the Raman C–D region.^57^ A similar approach would be required for the other biomolecule classes. Alternatively, spectral unmixing could be based on inhibiting specific enzymes responsible for fatty acid or protein biosynthesis and analyzing the difference spectra to the spectra of total deuterated biomass.^79^ A more simple approach was taken by Wang et al., who assigned the Raman bands centered at 2109, 2162, and 2215 cm^–1^ to deuterated lipids, proteins, and DNA, respectively.^54^ They used the according intensities as a rough estimation and explained their variations by metabolic differences.

Similarly, we described the C–D vibrations of biomass deuterated with glucose-d_12_ as a sum of three Gaussians using nonlinear least-squares fitting. The three model functions are centered at 2109 cm^–1^, 2160 cm^–1^, and 2220 cm^–1^, which we assigned to D-lipids, D-proteins, and D-DNA, respectively (Figure S5 and Table S1). These fits should be only regarded as rough estimations and the same boundary conditions (see Supporting Information for details on the data evaluation) have to be chosen for all fits that are compared. During the incubations with dPLA, the Raman bands differed significantly so that the C–D signature could not be sufficiently described by the same fitting procedure (Figure S4D). However, in Figure 3B the Raman bands for deuterated lipids, proteins and DNA are indicated by black dashed lines and show obvious variations in intensity. For all carbon-bound deuterium sources, D_2_O is formed as a result of catabolism. This can create locally high D_2_O concentrations directly inside the cells, which can then be taken up during other reactions. Nevertheless, this would only increase the overall biomass deuteration in the already involved cells without a preference for any specific synthesis pathway, and it cannot account for the observed intensity differences. Therefore, the C–D signature is anticipated to depend on the D source’s metabolic pathway, as previously shown by Wang et al.^54^

We assume that longer metabolic pathways, which consist of many enzymatically catalyzed steps where deuterium atoms might be replaced by hydrogen from water and other molecules, lead to a stronger dilution of the substrate’s deuterium label (see Figure S17 for a scheme of metabolic pathways). Looking at different substrate metabolisms, different deuteration degrees of biomolecule classes can be suggested.

If the bacteria have lactate dehydrogenase (as reported for a Sphingomonas wild strain^80^ and *E. coli*(81)) they could directly transform lactate into pyruvate, which is a precursor for acetyl coenzyme A (acetyl-CoA), the main C_2_-unit provider during chain elongation of fatty acid biosynthesis.^82^ On the other hand, not all amino acids, the building blocks for proteins and nucleotides/DNA, can be directly synthesized from pyruvate and more complex biosynthesis routes would be required.^83^ Therefore, stronger lipid than protein deuteration could be expected from the lactate metabolism.

If we now consider fatty acid biosynthesis from perdeuterated glucose, it will start with glycolysis, where nine enzymatically catalyzed steps lead to two pyruvate molecules as precursors for fatty acids. Due to the longer metabolic pathway, stronger dilution and lower lipid deuteration could be suggested than for lactate. Moreover, during glycolysis, intermediates can be introduced into other metabolic pathways necessary for amino acid synthesis, required for proteins and DNA. These pathways are more direct than starting by pyruvate, as discussed for the lactate metabolism, where pyruvate would require energetically demanding gluconeogenesis to form the intermediates required for syntheses of some amino acids (see Figure S17). Therefore, stronger protein deuteration could be suggested for the glucose instead of the lactate metabolism.

Indeed, compared to the glucose-d_12_ reference experiment, a lower relative D-protein to D-lipid content was observed with dPLA. This could be confirmed by analysis of a different region of the Raman spectrum where the bands of the phenylalanine (Phe) ring breathing vibrations are located (Figure S6), with the benzene stretching mode represented by a band at 1002 cm^–1^. This region is a particularly sensitive indicator of the degree of labeled protein because partial deuteration leads to the formation of isotopologues, as shown for the glucose-d_12_ reference so that the Phe ring breathing band at 1002 cm^–1^ was increasingly shifted to signals at 958 cm^–1^, 974 cm^–1^, and 986 cm^–1^ (in accordance with literature).^41,55^ Opposing this glucose-d_12_ reference experiment, after incubation with dPLA, only the nonlabeled and least labeled Phe isotopologues were found based on the Raman bands at 1002 and 958 cm^–1^ (Figure S6), evidencing low protein deuteration.

In contrast to the C–D Raman bands of D-protein and D-DNA of *S. koreensis* incubated with dPLA, the C–D vibrations of deuterated lipids could not be solely explained by the C–D signature of the glucose-d_12_ reference experiment. Here, a shift to higher wavenumbers was observed, and an additional C–D band at 2068 cm^–1^ emerged. Literature biomass spectra suggest that a reference experiment with D_2_O could shed light on this additional band since it was reported (although not discussed) for *Bacillus thuringiensis*(37) grown on glucose with 100% D_2_O and for *Streptococcus sanguis*(51) grown in Brain Heart Infusion medium with 50% D_2_O. Furthermore, similar Raman bands were previously reported for the CD_3_ symmetric stretching mode of a perdeuterated phospholipid,^84^ perdeuterated hexanoic acid,^85^ and other deuterated fatty acids.^57^ The additional 2068 cm^–1^ C–D band could thus originate from strongly deuterated lipids, as suggested in the discussion on the lactate metabolism.

To test this hypothesis, experiments with *S. koreensis* cells grown on nonlabeled glucose in 80% D_2_O were conducted to yield as high biomass and especially lipid deuterations as possible and thereby probe whether the reference spectra also show the 2068 cm^–1^ C–D band indicative of such a high lipid deuteration (Figure 3A,B (iv)). Indeed, a Raman band at 2068 cm^–1^ was observed for such high D_2_O concentrations, which supports its assignment to strong lipid deuteration (also see Figure S7, for lower D_2_O contents, which do not show this band). While the overall shape of the entire C–D signature in the D_2_O reference experiment resembled the one of the glucose-d_12_ reference, which is not surprising since the cells of both experiments were grown on glucose, the bands of the C–D vibrations of the dPLA experiment differed significantly (i.e., higher D-lipid and lower d-protein band relative intensities). This data can be explained with the hypothesized lactate metabolisms (see Supporting Information for more details), which would reinforce that dPLA is first depolymerized into its monomer, and lactate is then utilized by the bacteria.

An additional reference experiment was conducted with the perdeuterated dimer d,d-lactide-d_8_, which hydrolyzes to lactic acid in water. Although bacteria cultivated on deuterated and nondeuterated glucose showed no significant difference in their growth curves, bacteria previously grown on sodium-L-lactate were observed to display a long adaptation phase with little growth when subsequently incubated on d,d-lactide-d_8_ (Figure S8). Nonetheless, the cells harvested after 4 days showed some deuteration (Figure 3A,B (v)), with a similar C–D signature shape and an additional Raman band at 2068 cm^–1^ as observed in incubations with dPLA. These spectra, therefore, provide further support for detecting deuterated *S. koreensis* biomass in the dPLA incubation experiments, as evidenced by a stronger lipid and lower protein deuteration. The *E. coli* cells incubated with d,d-lactide-d_8_ showed the same C–D signature with elevated D-lipid content (Figure S9A). This spectroscopic analysis gives insights into the type of labeled biomolecules and, therefore, provides a mechanistic understanding that offers complementary data to mass-based methods like nanoSIMS. It not only helps to differentiate deuterated biomass from deuterated plastics but quantitative differences between lipid, protein, and DNA labeling can also be explained by differences in their biosynthesis pathways.

### Single-Cell Raman Spectra Unravel Phenotypic Heterogeneity and Temporal Changes During dPLA Incubation

**Figure 4:**
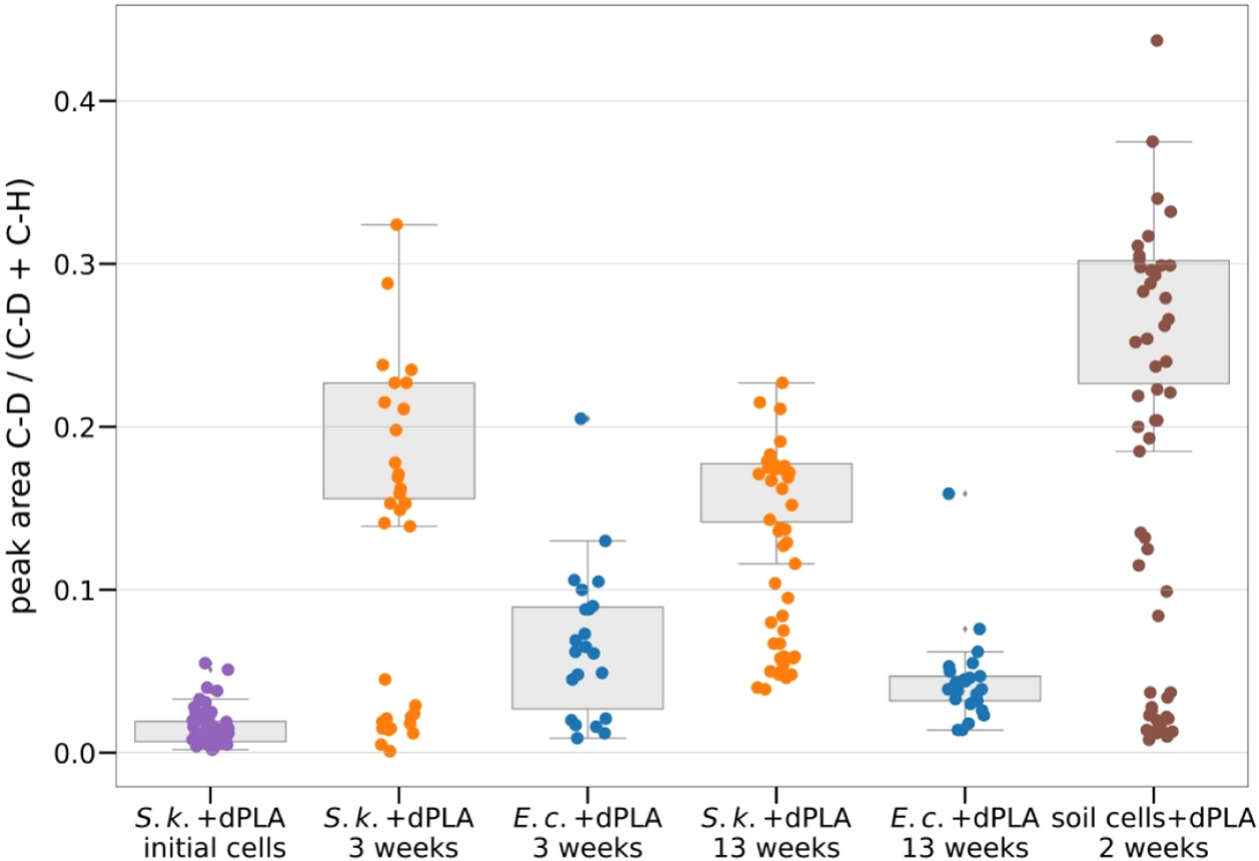
Temporal changes of single-cell deuterations during incubation with dPLA are determined based on the Raman band areas of the C–D over the C–D + C–H vibrations. Points depict single *S. koreensis* (S.k.) and *E. coli(E.c.)* cells or bacteria isolated from soil, and boxplots describe populations. Some Boxplots only describe the high deuteration clusters.

The overall deuteration degree of cells can be estimated by taking the ratio of the C–D band area (2050–2300 cm^–1^) over the sum of the C–D and C–H (2800–500 cm^–1^) band areas.^37,55^ However, noise and baseline in the Raman spectra affect the peak fitting, so even initially undeuterated cells may already (incorrectly) indicate a nominal deuteration of up to 5% (Figure 4) and are therefore considered as our baseline for the quantitative analysis. The parallel incubation of *E. coli* serves as a control for possible abiotic dPLA hydrolysis, which was previously reported for higher temperatures and lower pH.^17,19^ While the mean of the *S. koreensis* and *E. coli* populations incubated for 3 weeks with dPLA look similar, single-cell analysis revealed a bimodal distribution of highly deuterated (between 14 and 29% deuteration) and undeuterated (below 5%)/nonactive cells within the *S. koreensis* population (Figure 4). Since the sample was mixed with a vortexer before a subsample was taken for the analysis, the deuteration difference could be explained by one cluster of biofilm cells, strongly exposed to the plastic, versus a second cluster of planktonic cells,^86^ which have not been in contact with the plastic. Calabrese et al. reported high initial anabolic heterogeneity in isogenic populations under nutrient-replete conditions, which decreased over time and suggested differences in metabolic fitness and history to be the cause.^87^

Similarly, the initially strong phenotypic heterogeneity within the *S. koreensis* population could alternatively be explained by differences in metabolism optimization toward the new substrate based on the cell’s metabolic fitness. Variations in metabolic fitness have been previously shown for *S. koreensis* grown on glucose in deuterated water without changing the initial carbon source.^38^ On the contrary, *E. coli* cells showed lower deuterations of up to 14% after 3 weeks of incubation with dPLA. In the reference experiment with d,d-lactide-d_8_, *E. coli* yielded higher growth rates than *S. koreensis* and incorporated more deuterium (Figures S8 and S9). This suggests faster metabolism optimization. However, the deuteration levels after incubation with dPLA are relatively low, which indicates that deuterium is readily available in low quantities and some cells are either not active or not exposed to the deuterium source. While H-D exchange is plausible for the dPLA deuterium in the α position to the carbonyl group based on keto–enol tautomerism, it cannot account for the observed biomass deuteration for two reasons. First, the total deuterium in dPLA only accounts for less than 1% of the entire hydrogen pool in the incubation experiments (see Supporting Information for calculations), which cannot lead to such a high biomass deuteration if taken up by H-D exchange through water. Second, in case of H-D exchange, the deuterium would be taken up in the form of D_2_O, leading to a different Raman C–D band shape, as demonstrated in experiments with deuterated water (Figure S7). Instead, the band shape resembles the one for incubation with d,d-lactide-d_8_ (Figures S9 and S10). Considering the likely hydrolysis of lactide to lactic acid in water, this suggests a metabolic pathway based on the uptake of deuterated monomer.

Size exclusion chromatography of dPLA showed a high average molar mass (*M*_n_ = 3900 g/mol) and a low polydispersity (*M*_w_/*M*_n_ = 1.16) with no measurable traces of oligomers (Figure S16). In the polymerization experiment, the monomer consumption was almost quantitative, and small monomer residues would have been removed in the purification process after polymerization. Thus, d,d-lactide-d_8_ monomer residues or low molecular weight oligomers cannot account for the extent of the readily available D source during biodegradation experiments, and breakage of the ester bond in the polyester backbone (Figure S1) is suggested.

PLA is known to slowly hydrolyze in the presence of water.^13,17−19,88^ While higher temperatures and acidic environments accelerate hydrolysis, only very slow abiotic hydrolysis is expected at 21 °C and a pH of 7. Amorphous polymer regions have been reported to hydrolyze first.^18,89,90^ Although the used dPLA is expected to be of high crystallinity, there are always some amorphous regions and the low deuteration of *E. coli* cells could thus originate from the uptake of hydrolysis products of these regions. We can derive a threshold for the readily available D source based on the maximum deuteration of the *E. coli* biomass controls.

Although the cluster of the strongly labeled *S. koreensis* cells (56% of the cells) slightly decreased after 13 weeks of incubation with dPLA, it still lay above the *E. coli* threshold, suggesting additional biotic dPLA depolymerization and microbial uptake. Initially, the dPLA concentration was 0.3 g/L, and the catabolic breakdown of dPLA led to an enhanced deuterium incorporation into the *S. koreensis* biomass. However, after 13 weeks of incubation, slightly less deuteration was observed in the biomass, which could indicate that the bioavailability of dPLA decreased. As dPLA was the only added carbon source, it can be assumed that bacterial growth was attenuated, and cells transitioned into stationary phase. There, hydrogen may be incorporated from the aqueous medium during the turnover of biomolecules, leading to a dilution of the previously incorporated D-label. Here, complementary information on D_2_O production may be gained by measuring isotope values in water as an indicator of additional D_2_O input. As shown in Figure 4, despite this subtle dilution of the biomass label by hydrogen from water, SIRM still allowed tracing D from labeled PLA into microbial biomass based on characteristic C–D vibrations. The approach, therefore, holds great promise for qualitatively detecting and analyzing this step of plastic biodegradation.

To put the biomass deuteration of the dPLA incubations into context, perdeuterated glucose was chosen as an alternative carbon-bound D source and mixed with glucose at natural isotopic abundance to create different deuteration ratios (Figure S11). The uptake of the D-label increased with higher D availability in the medium and reached deuterations of up to 40% for growth on 100% glucose-d_12_. A similarly high deuteration of up to 30% was reported for *P. putida*, grown on perdeuterated glucose.^55^ Despite the dilution of the label by H_2_O, even 20% deuterated glucose (minor label applied in reference experiments) led to statistically significant labeling (Figure S11, Student’s *t* test: 8.33 with *p* < 0.001). While the C–D band area of the mean spectrum of *S. koreensis* incubated for 3 weeks with dPLA appeared to lie between that in 20 and 50% glucose-d_12_ reference experiments (Figure S11A), the single cell spectra (Figure S11B) reveal that one part of the population remained undeuterated, while the rest achieved deuterations close to the 70% deuterated glucose reference. This highlights the importance of single-cell analysis.

### Deuteration of Soil Bacteria with dPLA Promotes the Generalizability of the Method

With the intention of adapting the soil microbiome to PLA, the soil had previously been incubated with commercially available PLA and was subsequently exposed to perdeuterated PLA. Indeed, already after 2 weeks of incubating the isolated microbial consortium with dPLA, strong biomass deuterations (Figure 4) with a similar C–D signature as with *S. koreensis*/dPLA incubations were observed (see Figure S12 for a mean Raman spectrum). In contrast to monospecies incubations, the cells can be classified into three distinct clusters according to their degree of deuteration. The nondeuterated cluster likely represents inactive cells, cells not exposed to the polymer or species not able to metabolize the depolymerization products. The high deuteration cluster likely represents cells that are directly involved in the biodegradation process of dPLA. The third cluster with low deuterations could be explained either as feeding from the same deuteration source as the *E. coli* cells without being actively involved in the dPLA depolymerization or by cross-feeding from cells that are actively involved in dPLA biodegradation. This shows that our approach can also be transferred to more complex systems.

### Carotenoids as Tracers for *Sphingomonas koreensis* Deuteration During Incubation with dPLA

**Figure 5:**
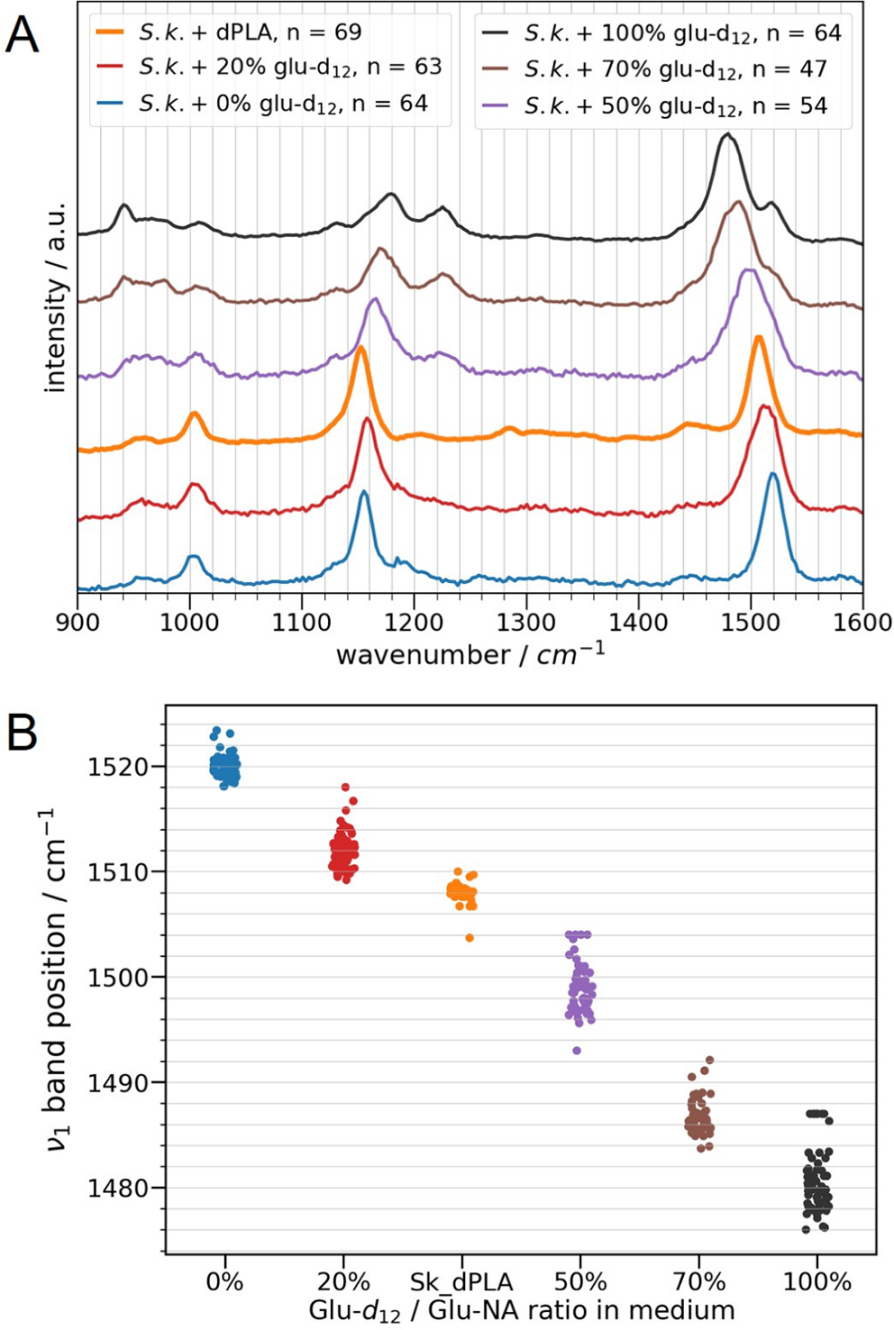
(A) Resonance Raman spectra of a number of n *S. koreensis* cells incubated with 0, 20, 50, 70, and 100% glucose-d_12_ are compared to the mean spectrum of 69 cells incubated with dPLA. (B) The ν_1_-band position of the single-cell spectra is plotted against the glucose-d_12_ ratio. A lower band position reflects a higher deuterium content.

If a chromophore system (such as in carotenoid pigments) has an electronic transition close to the energy of the excitation wavelength (e.g., 532 nm), it shows by order of magnitude increased Raman scattering. In Resonance Raman spectroscopy, this effect offers the advantage of using lower laser powers and shorter integration times. Further, it makes some colored compounds detectable despite their relatively low molar abundance. Resonance Raman spectra of *S. koreensis* cells showed three predominant carotenoid bands at 1521 cm^–1^, 1156 cm^–1^, and 1005 cm^–1^ (Figure 5A), which can be assigned to the C=C stretching (ν_1_), C–C stretching (ν_2_), and CH_3_ rocking (ν_3_) vibrations, respectively.^38,69,91^ Raman bands of conventional biomass Raman spectra are much lower in intensity and, therefore, buried in the background.

To use carotenoids as tracers of *S. koreensis* deuteration, reference spectra with known glucose-d_12_ ratios (Figure 5A) were measured. Although no H atoms are directly involved in the ν_1_ and ν_2_ vibrations, deuteration leads to different shifts in the wavenumbers of the C=C and C–C stretching vibrations. A higher D content leads to a pronounced red shift of the ν*_1_*-band, while a shoulder responsible for undeuterated biomass remains at the original ν_1_-band position. The ν_2_-band is split upon deuteration, and both resulting peaks are apparently blue-shifted. These trends are explained by the previous coupling of the ν_1_ and ν_2_ stretchings to C–H bendings (1200–1300 cm^–1^) for the undeuterated molecules. In the case of deuteration, the C–H bendings are red-shifted to wavenumbers below 1000 cm^–1^ and thereby decoupled from the ν_1_ and ν_2_ vibrations, leading to the Raman bands of uncoupled C=C and C–C stretching vibrations, which appear red- (C=C) and blue-shifted (C–C) compared to the initial, coupled vibrations for the undeuterated molecules (see Supporting Information for more details).^38,69^

In our experiments, the observed red shift of the ν_1_-band by 41 cm^–1^ (1521 to 1480 cm^–1^) indicated a stronger deuteration with glucose as a D source (Figure 5) compared to the literature where D_2_O was used,^38^ which makes carotenoids a promising tracer. A more substantial shift of 64 cm^–1^ was reported for perdeuterated carotenoids isolated from *Chlorella* sp.^69^ The blue shifts of the ν_2_ vibrations differed strongly for all three systems (*S. koreensis* + glucose-d_12_, *S. koreensis* + D_2_O,^38^*Chlorella* sp. + D_2_O^69^), and they are not further discussed here. Instead, the ν_1_ band position was used to trace the carotenoid deuteration. Daily wavenumber corrections based on a silicon wafer allowed to compare absolute band positions. The most intense ν_1_ Raman band was fitted with a Gaussian profile, and the band positions of the single spectra are shown in Figure 5B. Although the initial cell suspensions of *S. koreensis* incubated with dPLA showed no carotenoids, they were detected after 17 weeks. In other experiments with simple substrates, we also observed delayed carotenoid production, which could be caused by several environmental factors, i.e., UV light, temperature, pH or nitrogen deficiency. The corresponding ν_1_ band position suggests significant deuteration, which lies between the deuteration of the reference spectra of the incubation experiments with 20 and 50% glucose-d_12_ (Figure 5). Thereby, D is traced from dPLA into carotenoids as part of the *S. koreensis* biomass. Since a maximal ν_1_ band shift of 24 cm^–1^ was reported for labeling with 90% D_2_O,^38^ we can again rule out that H-D exchange from dPLA into the medium is responsible for the deuteration. Although resonance Raman spectroscopy only gives information about one type of biomolecule within the cell, qualitative and quantitative information can be gained with greater precision. These advantages could facilitate Raman imaging applications for the analysis of microbial degradation of microplastics, where short acquisition times and high Raman intensities are favorable. Resonance Raman spectroscopy, hence, proved to be a reliable, fast, and simple alternative to conventional Raman spectroscopy.

## Conclusions

Stable isotope Raman microspectroscopy holds significant potential to study the fate of polymers such as polylactic acid, a terrestrial and aquatic pollutant, under environmentally relevant conditions (pH and temperature). The approach directly traces a deuterium label from plastic into distinct biomolecules (lipids, proteins, DNA, and carotenoids) of microbial biomass at the single-cell level. In comparison with controls (experiments with *E. coli* and different D sources), plastic biodegradation can be delineated. Where bulk methods would have only reported the mean of the *S. koreensis* population, which is similar to that of the *E. coli* control, a bimodal single-cell distribution was observed with SIRM. Furthermore, variations in the C–D signatures of *S. koreensis* cells incubated with dPLA, as opposed to other deuterium sources, can be traced to differences in the lactate and glucose metabolism as likely underlying driver. The approach was transferred to a microbial community isolated from soil, where three clusters with different microbial deuterium incorporations were observed. In follow-up studies, the approach could be established with other perdeuterated polymers or applied to terrestrial samples without transferring the microbiome to an artificial medium. Due to abundance of D and synthesis pathways, deuterated compounds are generally cheaper and often easier available than ^13^C-labeled ones, which make them more promising for such experiments or future field studies that require a certain amount of labeled substrate.

Moreover, our approach is a valuable tool to investigate the effect of different parameters on the biodegradability of plastics. For example, potential variations in the ratio of deuterated lipids, proteins, and DNA based on environmental conditions (e.g., temperature) or nitrogen availability may be explored. The broadened mechanistic understanding could thus help to remediate environments with high plastic exposure and to design new biodegradable polymers with shorter degradation times.

## Supporting Information Available

Chemical structure of d,d-dPLA, single Raman spectra used for mean spectra, analysis of Phe deuteration, growth curves, Raman spectra of incubations with d,d-lactide-d_8_, glucose-d_8_ and D_2_O, and dPLA SEC curve and SEM images plus interpretation, more experimental details, a summary of the data processing procedure, calculations on the estimated D content in the medium in the hypothetical case of H–D exchange, and more details on suggested glucose and lactate metabolisms and the apparent blue-shift of the carotenoid band (PDF)
