# Supplementary material for: Raman Microspectroscopy to Trace the Incorporation of Deuterium from Labeled (Micro)Plastics into Microbial Cells
Source: Anal Chem. 2025 Feb 10;97(8):4440–51. doi: 10.1021/acs.analchem.4c05827 (PMC11883734; doi:10.1021/acs.analchem.4c05827)
Supplement: Supplementary file 1 — ac4c05827_si_001.pdf [file ac4c05827_si_001.pdf]

Supporting Information

Raman Microspectroscopy to Trace the Incorporation of Deuterium from Labeled (Micro)Plastics into Microbial Cells

Kara Müller[1], Martin Elsner[1], Anna E. Leung[2], Hanna Wacklin-Knecht[2,3], Jürgen Allgaier[4], Maria Heiling[5], Natalia P. Ivleva[1]*

[1] Chair of Analytical Chemistry and Water Chemistry, School of Natural Sciences, Technical University of Munich, Lichtenbergstr. 4, 85748 Garching, Germany.

[2] Scientific Activities Division, European Spallation Source ERIC, Lund 221 00, Sweden.

[3] Division of Physical Chemistry, Department of Chemistry, Lund University, P.O. Box 124, SE-22100 Lund, Sweden.

[4] Jülich Centre for Neutron Science (JCNS-1), Forschungszentrum Jülich GmbH, 52428 Jülich, Germany.

[5]Soil and Water Management and Crop Nutrition Laboratory, Joint FAO/IAEA Division of Nuclear Techniques in Food and Agriculture, International Atomic Energy Agency (IAEA), Vienna, Austria.

Table of Contents

Table of Figures S3

Additional Figures S5

Expansion of Materials and Methods section S12

Washing of bacterial cells S12

Soil microcosm experiments S13

Bacteria isolation from soil samples S13

Scanning electron microscopy (SEM) of dPLA particles S13

Size-exclusion Chromatography (SEC) of dPLA S16

Data processing S17

Mean spectra: S17

Quantitative analysis – C-D and C-H Raman bands: S17

Quantitative analysis – Resonance Raman spectra: S19

Extended discussion on different plausible biosynthesis pathways of amino acids and fatty acids with either lactate or glucose as substrate S20

Lactate as substrate S20

Glucose as substrate S21

Estimation of deuterium content in the biodegradation experiments S22

An extended explanation of apparent isotope shifts of carotenoid Raman bands upon deuteration S22

References S23

Table of Figures

Figure S 1 Chemical formular of stereodefined perdeuterated polylactic acid (D,D-dPLA)........................S5

Figure S 2 Examples of fits (red) of overlapping Sphingomonas koreensis cells with dPLA particles to Raman spectra of S. koreensis cells incubated with dPLA (blue). Raman difference spectra are shown in grey. S5

Figure S 3 Reproducibility of single cell spectra of S. koreensis cells, incubated for (A) 3 or (B) 13 weeks with dPLA. S6

Figure S 4 The C D region (2020 – 2300 cm^{-1}) of initial dPLA particles (A) and dPLA particles incubated with S. koreensis for 20 weeks (B). The Raman band at 2070 cm^{-1} of all spectra in (A) and (B) is fitted with a Gaussian function, and the bandwidth is given in (C). As a comparison, the bandwidth of the 2068 cm^{-1} peak in the mean spectrum of S. koreensis cells incubated with dPLA is 12 cm^{-1} (D). All Raman spectra have the wavenumbers in the region of 2020 – 2300 cm^{-1} as x-axis. S6

Figure S 5 Three Gaussian fits can describe the C H and C D vibrations of S. koreensis, incubated with 100 % glucose-d_{12}. The model functions are associated with lipids, proteins, and DNA. S7

Figure S 6 Mean Raman spectra of S. koreensis cells incubated with 0, 20, 50, 70, and 100 % glucose-d_{12} in the wavenumber range of the original phenylalanine band and its isotopologues. Additionally, the mean Raman spectrum of S. koreensis incubated with dPLA for 3 weeks is included. S8

Figure S 7 C-D signatures of mean Raman spectra of S. koreensis cells incubated with different D_2O ratios. The Raman band at 2068 cm^{-1}, indicative of strong lipid deuteration, appears for D_2O contents of 50% and above...S8

Figure S 8 Growth curves in three to four replicates of S. koreensis (A) and E. coli (B) with deuterated (red) and non-deuterated D-glucose (black), D,D-lactide-d_8 (green), and sodium-L-lactate (blue). A negative control is included (purple), where no organic carbon was supplemented in the growth medium. This medium was also used to wash the cells three times before they were transferred from the sodium-L-lactate medium to the new medium. S9

Figure S 9 A. Mean Raman spectra of S. koreensis and E. coli incubated with either D,D-lactide-d_8 or glucose-d_{12} in separate experiments. B. C-D peak area ratios are shown for single cells. The colors are the same as in A. S9

Figure S 10 Mean Raman spectrum of 22 E. coli cells incubated with dPLA for 3 weeks. S10

Figure S 11 A. Mean Raman spectra of S. koreensis (S.k.) incubated with different ratios of glucose-d_{12} / glucose-d_0 are compared to the mean spectrum of all S. koreensis cells measured after three weeks of incubation with dPLA (orange). B. The C-D / (C-D + C-H) peak area ratios are calculated for the single cells. While all populations of the glucose-d_{12} references show an increase in the peak area ratio, the population of the dPLA incubation is divided into two clusters – one undeuterated and one similar to the 70 % glucose-d_{12} reference..S10

Figure S 12 Mean Raman spectrum of 54 bacterial cells from soil consortium incubated for two weeks with dPLA in minimal medium. S11

Figure S 13 Scanning electron microscopy images of a dPLA suspension in MilliQ water. The images were obtained with 500x magnification, a voltage of 1 kV, and an aperture of 30 µm with a secondary electron microscope. The scale bars in the lower right corner of the images correspond to 20 µm. The particles which were measured for the size distribution are marked with a yellow line. S14

Figure S 14 Scanning electron microscopy images of a dPLA suspension in MilliQ water. The sample was sputtered with Pt to increase the conductivity. The images were obtained with 10.000x magnification, a voltage of 5 kV, and an aperture of 30 µm with a secondary electron microscope. The scale bars in the lower right corner of the images correspond to 1 µm. The particles which were measured for the size distribution are marked with a yellow line. S15

Figure S 15 Deuterated PLA particles size distributions were roughly estimated from the SEM images in Figures S8 & S9. A. 115 large particles were measured under 500x magnification. B. 231 sputtered, smaller particles were measured under 10.000x magnification. S15

Figure S 16 SEC RI-trace of dPLA. The number average molecular weight (Mn) of the dPLA sample is Mn = 3900 g/mol with narrow molecular weight distribution (Mw/Mn = 1.16, with Mw being the weight average molecular weight). S16

Figure S 17 Metabolic pathways from glucose and lactate to fatty acids and amino acids . Glucose undergoes glycolysis and serves as a precursor for the synthesis of all the depicted amino acids and fatty acids. In contrast, the conversion of lactate into pyruvate indicates a preferential pathway for the synthesis of fatty acids and the amino acids shown in the dark blue boxes. To produce other amino acids, such as phenylalanine, pyruvate must first enter the gluconeogenesis pathway, which is an energy-intensive process. S20

Additional Figures

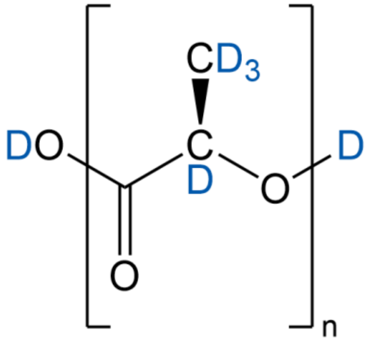

stereodefined, perdeuterated
polylactic acid (D,D-dPLA)

Figure S 1 Chemical formular of stereodefined perdeuterated polylactic acid (D,D-dPLA).

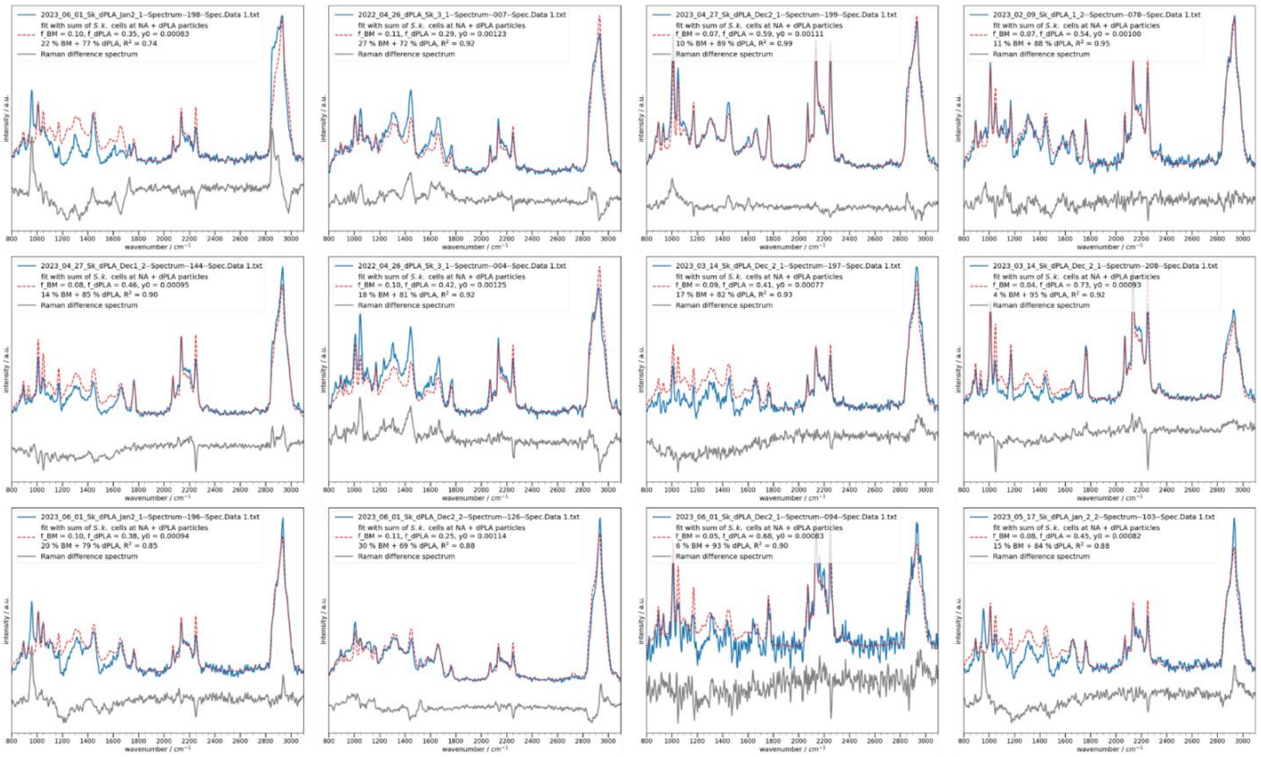

Figure S 2 Examples of fits (red) of overlapping Sphingomonas koreensis cells with dPLA particles to Raman spectra of S. koreensis cells incubated with dPLA (blue). Raman difference spectra are shown in grey.

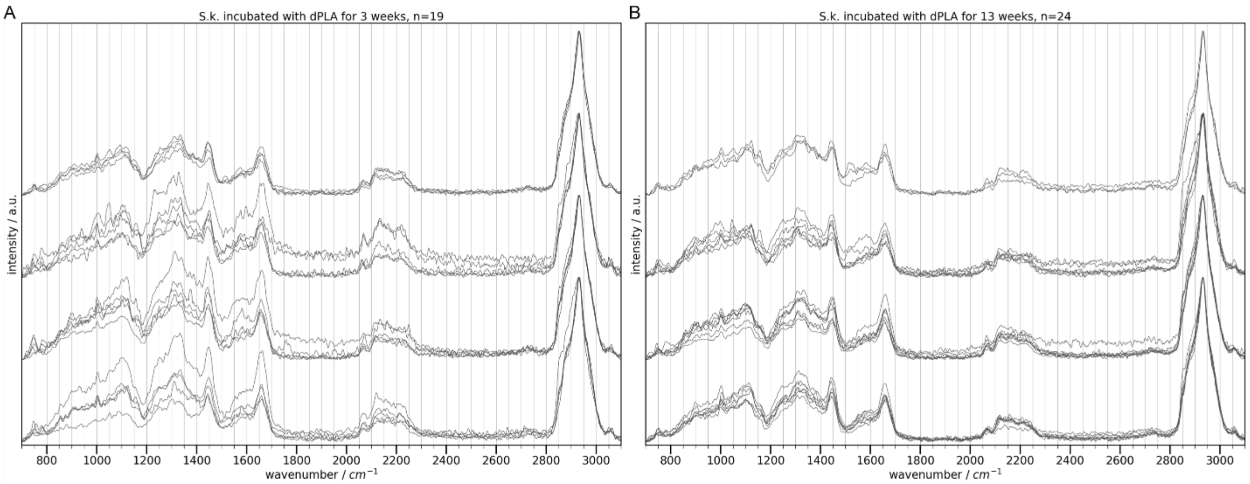

Figure S 3 Reproducibility of single cell spectra of S. koreensis cells, incubated for (A) 3 or (B) 13 weeks with dPLA.

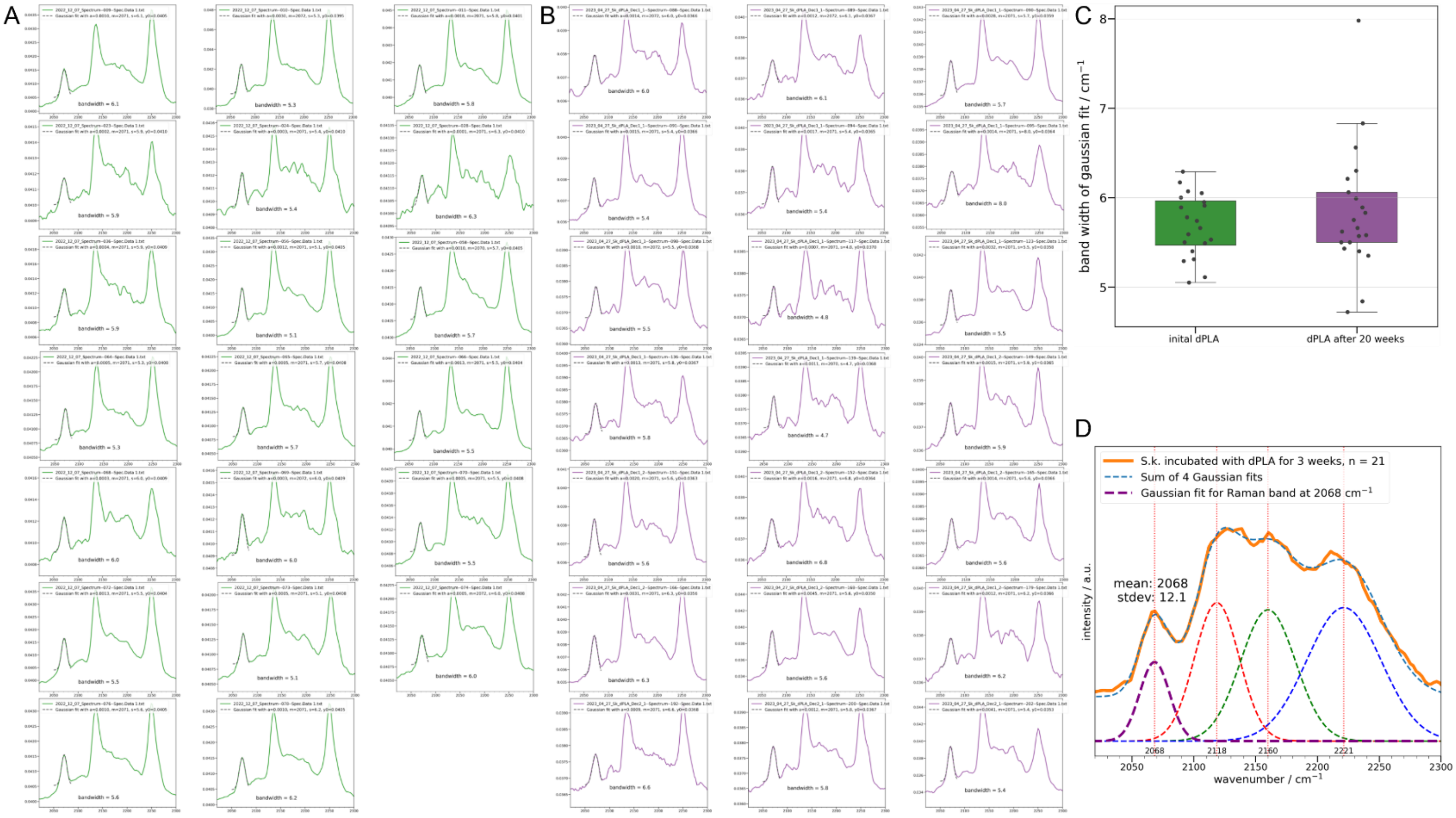

Figure S 4 The C D region (2020 – 2300 cm^{-1}) of initial dPLA particles (A) and dPLA particles incubated with S. koreensis for 20 weeks (B). The Raman band at 2070 cm^{-1} of all spectra in (A) and (B) is fitted with a Gaussian function, and the bandwidth is given in (C). As a comparison, the bandwidth of the 2068 cm^{-1} peak in the mean spectrum of S. koreensis cells incubated with dPLA is 12 cm^{-1} (D). All Raman spectra have the wavenumbers in the region of 2020 – 2300 cm^{-1} as x-axis.

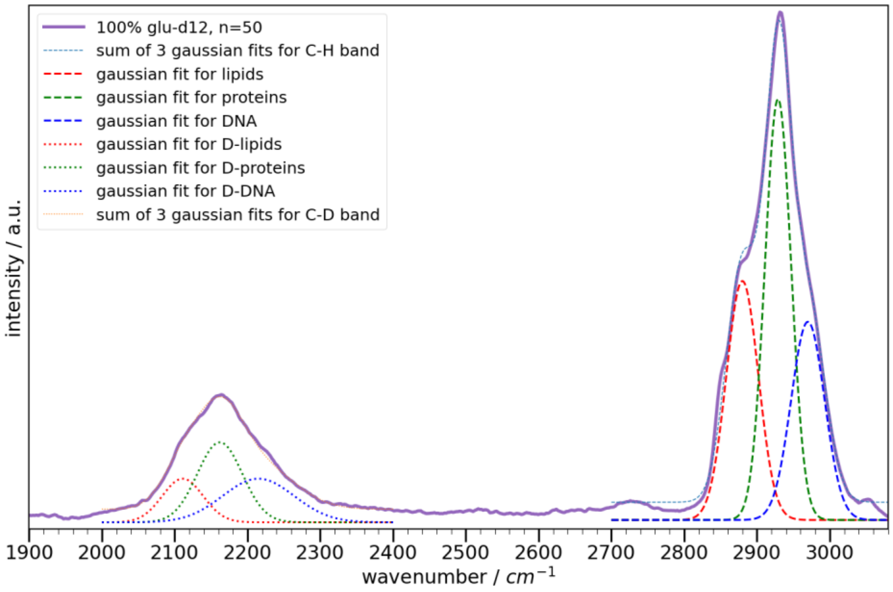

Figure S 5 Three Gaussian fits can describe the C H and C D vibrations of S. koreensis, incubated with 100 % glucose-d$_{12}$. The model functions are associated with lipids, proteins, and DNA.

Table 1. Raman band positions and their peak area ratios of lipids, proteins, and DNA in Figure S4 is compared to literature.

Band assignment	**C-H**				**C-D**			
	here		Wang et al.[1]		here		Wang et al.[1]	
**lipids**	2880	31%	2874	27%	2109	25%	2109	32%
**proteins**	2929	40%	2928	42%	2162	36%	2160	35%
**DNA**	2970	29%	2962	31%	2215	35%	2220	33%

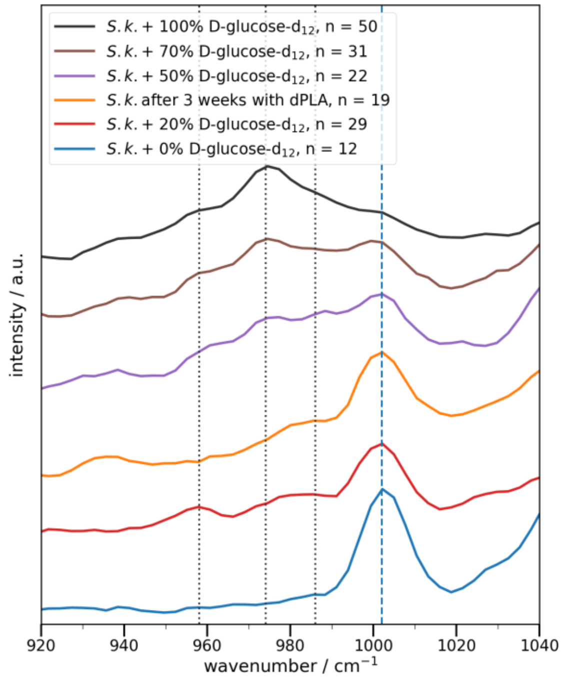

Figure S 6 Mean Raman spectra of S. koreensis cells incubated with 0, 20, 50, 70, and 100 % glucose-d_{12} in the wavenumber range of the original phenylalanine band and its isotopologues. Additionally, the mean Raman spectrum of S. koreensis incubated with dPLA for 3 weeks is included.

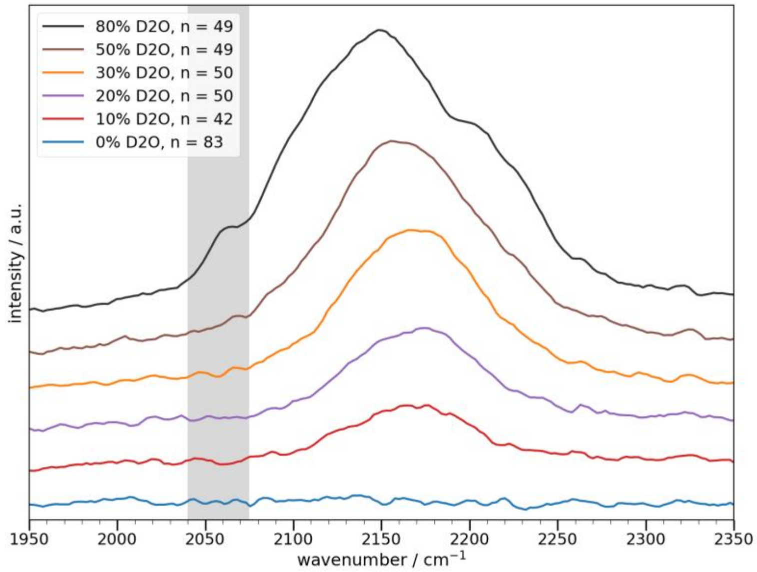

Figure S 7 C-D signatures of mean Raman spectra of S. koreensis cells incubated with different D_2O ratios. The Raman band at 2068 cm^{-1}, indicative of strong lipid deuteration, appears for D_2O contents of 50% and above.

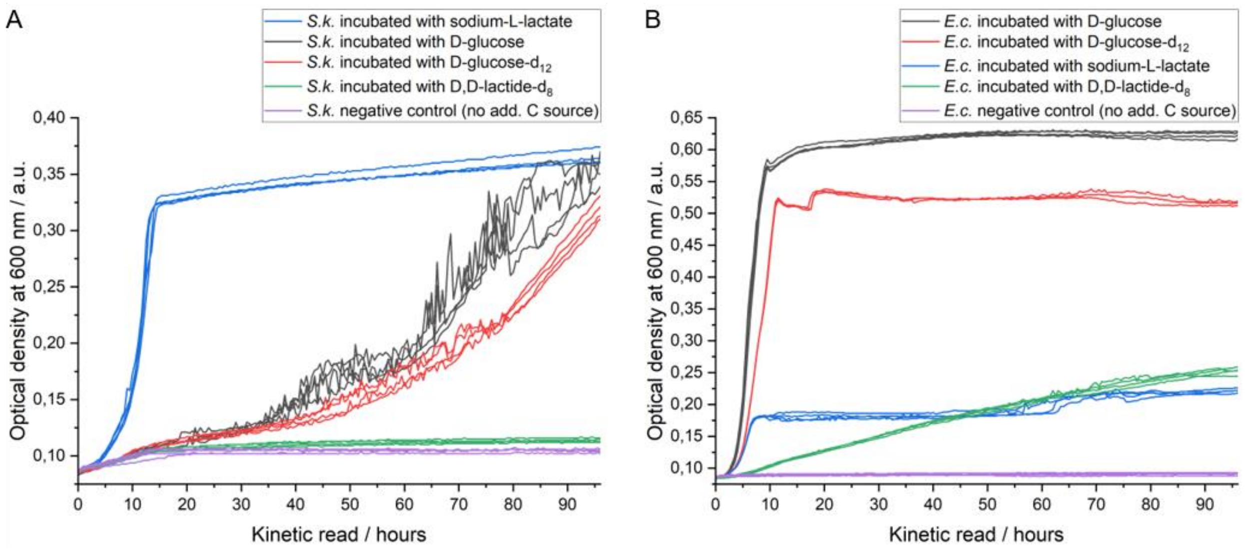

Figure S 8 Growth curves in three to four replicates of S. koreensis (A) and E. coli (B) with deuterated (red) and non-deuterated D-glucose (black), D,D-lactide-d_8 (green), and sodium-L-lactate (blue). A negative control is included (purple), where no organic carbon was supplemented in the growth medium. This medium was also used to wash the cells three times before they were transferred from the sodium-L-lactate medium to the new medium.

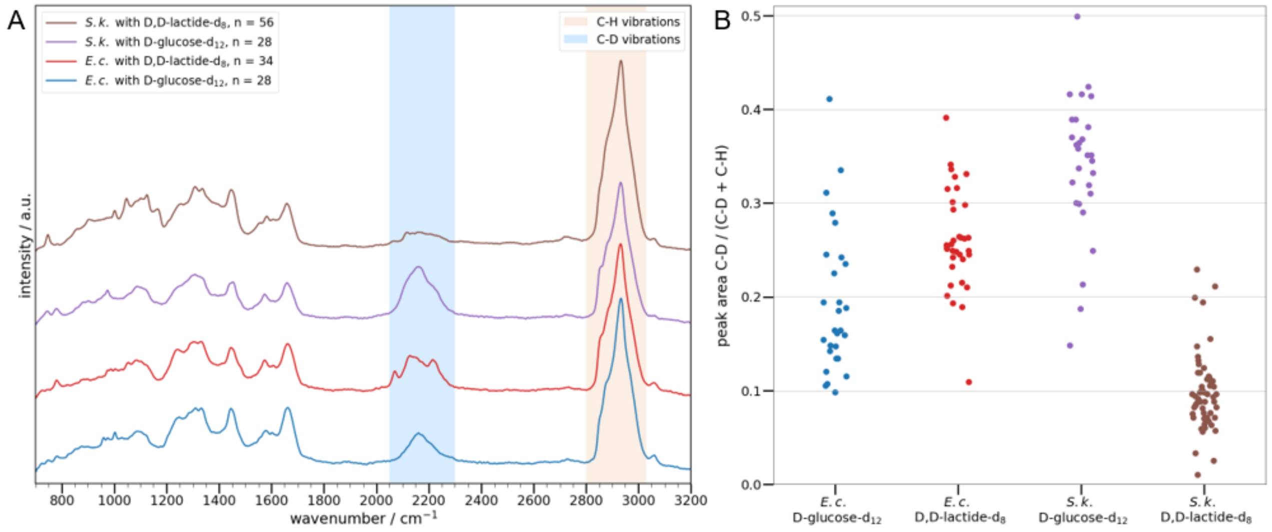

Figure S 9 A. Mean Raman spectra of S. koreensis and E. coli incubated with either D,D-lactide-d_8 or glucose-d_{12} in separate experiments. B. C-D peak area ratios are shown for single cells. The colors are the same as in A.

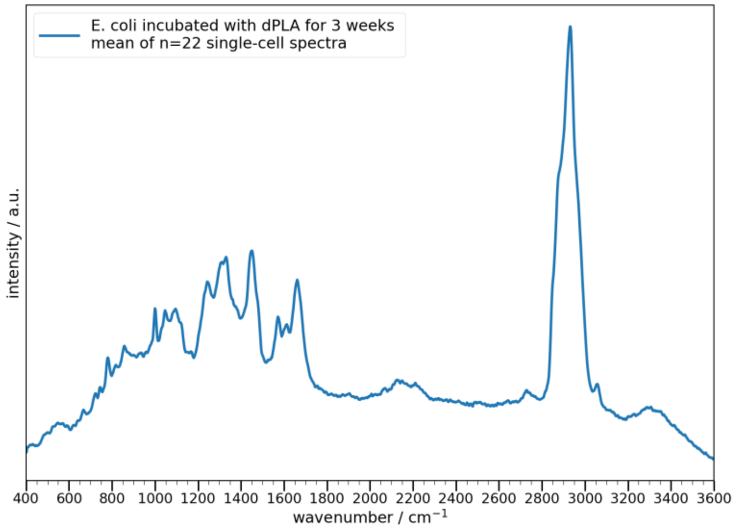

Figure S 10 Mean Raman spectrum of 22 E. coli cells incubated with dPLA for 3 weeks.

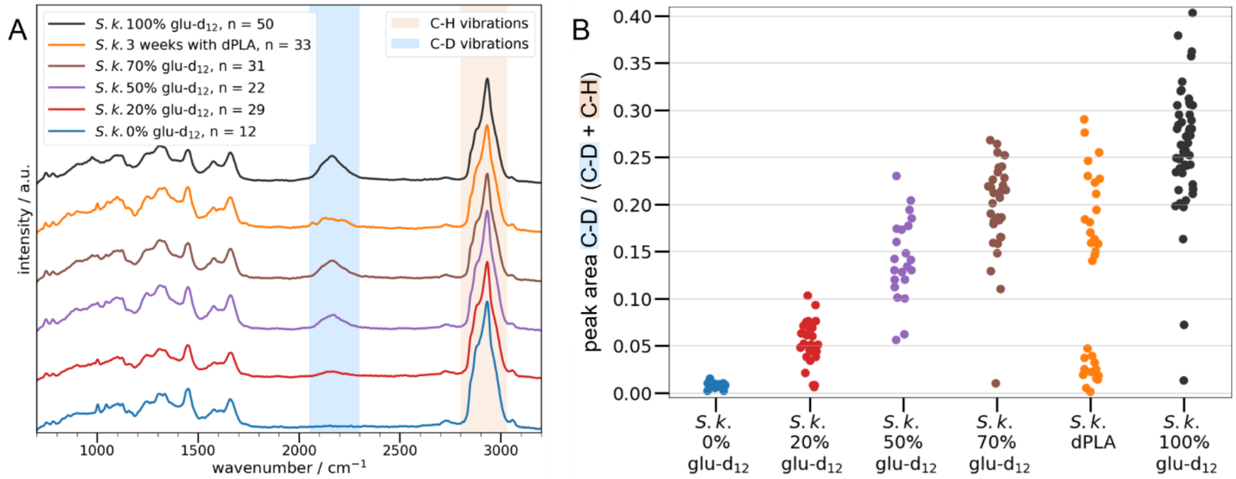

Figure S 11 A. Mean Raman spectra of S. koreensis (S.k.) incubated with different ratios of glucose-d_{12} / glucose-d_0 are compared to the mean spectrum of all S. koreensis cells measured after three weeks of incubation with dPLA (orange). B. The C-D / (C-D + C-H) peak area ratios are calculated for the single cells. While all populations of the glucose-d_{12} references show an increase in the peak area ratio, the population of the dPLA incubation is divided into two clusters – one undeuterated and one similar to the 70 % glucose-d_{12} reference.

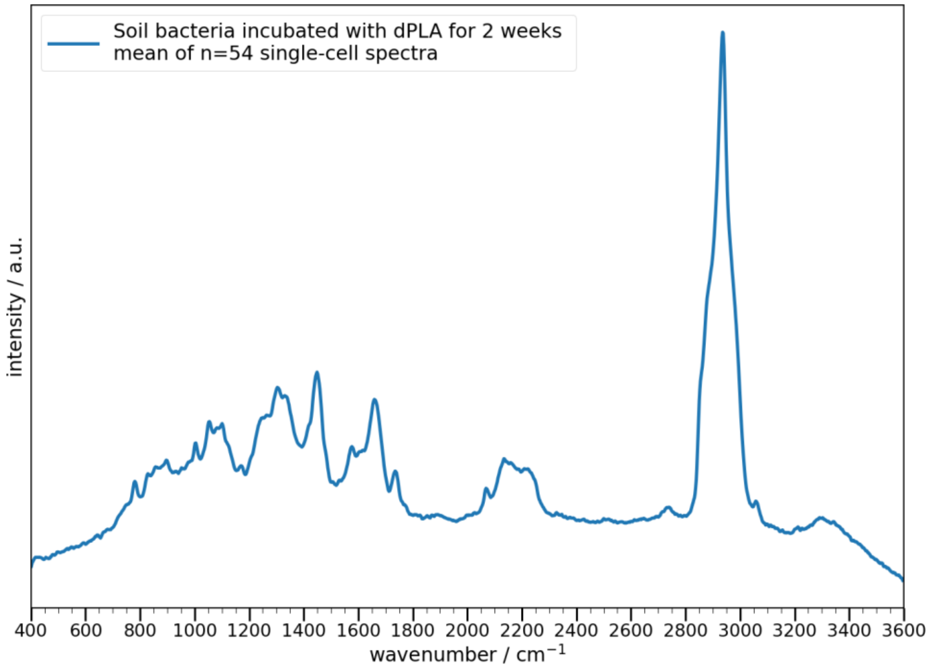

Figure S 12 Mean Raman spectrum of 54 bacterial cells from soil consortium incubated for two weeks with dPLA in minimal medium.

Expansion of Materials and Methods section

Washing of bacterial cells

Before a medium was changed (e.g. to change the carbon source), cells were washed three times in the new medium according to the following description. Based on the same procedure, cells were washed three times in MilliQ water before Raman measurement.

The cell suspension was centrifuged at 6000 rpm for three minutes. The supernatant was discarded, and the cell pellet was resuspended in the new medium with double the volume of the initial suspension. This step was repeated two times, and the final cell pellet was resuspended in the initial volume of medium.

Reference incubation experiments of *Sphingomonas koreensis* with different deuterium sources:

Table 2. Description of *S. koreensis* reference experiments (MM: optimized minimal medium according to Weng et al., NA: natural isotopic abundance)

Carbon source	Medium	Aim of the experiment
D-glucose-d_{12}<br>(4 g/L, perdeuterated)	MM (NA)	Reference of biomass deuterated with carbon-bound D source
D-glucose<br>(4 g/L, NA)	MM<br>with 80% D_2O	Reference of biomass with as high deuteration as possible to check for C-D Raman band of strongly deuterated lipids at 2068 cm^{-1}
D,D-lactide-d_8<br>(4 g/L, perdeuterated)	MM (NA)	Reference of biomass deuterated with the dimer of the used polymer. In case of biodegradation the polymer is first depolymerized and the mono-, di- and oligomers are then metabolized by the bacteria. So this reference should be similar to the spectra obtained in the dPLA biodegradation experiment.
Different ratios of D-glucose-d_{12} and D-glucose at NA (total concentration always 4 g/L)	MM (NA)	Reference of different deuteration levels to see linear correlation and to quantitatively compare the deuteration levels obtained in the dPLA biodegradation experiment with a reference.

Soil microcosm experiments

Table 3. Soil characteristics of Grabenegg soil, as sampled in 2022 at a depth of 0 –15 cm was sieved to 2 mm and used for incubations with conventional PLA (Ingeo™ Biopolymer 2003D, NatureWorks LLC). Bacteria were isolated from soil incubation samples in 2024.

Parameter	Result	Unit	Evaluation
pH: $CaCl_2$	6,8		neutral
Calcium carbonate ($CaCO_3$)	<0,5	%	low
Phosphorous (P): CAL	35	mg/kg	low
Potassium (K): CAL	54	mg/kg	low
Humus content	2.0	%	humous
Total nitrogen (N)	0.142	%	normal 0.10 – 0.25 %
Nitrogen mineralization potential	81	mg/kg/7d	high
Sand	36.8	%	
Silt	50.0	%	
Clay	13.2	%	light
Calcium (Ca): exchangeable	11.20	cmolc/kg	85.7 % Ca of sorption complex
Magnesium (Mg): exchangeable	1.55	cmolc/kg	11.9 % Mg of sorption complex
Potassium (K): exchangeable	0.22	cmolc/kg	1.7 % K of sorption complex
Sodium (Na): exchangeable	0.08	cmolc/kg	0.6 % Na of sorption complex
Aluminum (Al): exchangeable	<0.06	cmolc/kg	0 % Al of sorption complex
Iron (Fe): exchangeable	<0.00	cmolc/kg	0 % Fe of sorption complex
Manganese (Mn): exchangeable	0.02	cmolc/kg	0.1 % Mn of sorption complex
H-value	0.002	cmolc/kg	% protons of sorption complex
Exchange capacity	13.07	cmolc/kg	Normal: 10-40 cmolc/1000g

Bacteria isolation from soil samples

7.2 g of soil was homogenized in 22.5 mL sterile-filtered phosphate-buffered saline (PBS) by shaking the sample tube with an overhead shaker for 30 minutes. 85 mg polyvinyl pyrrolidine and 31 mg $Na_4P_2O_7 \cdot 10H_2O$ and 117µL Tween20 were added to each sample, which was mixed again with an overhead shaker for 135 minutes. 22.5 mL of Nycodenz (Progen Biotechnik, Heidelberg, Germany) solution with a density of 1.42 g/mL (stock solution: 92 g Nycodenz in 71 mL sterile-filtered MilliQ water) were provided on the bottom of a vial and the soil slurry was gently poured on top of it to retain the two phases. The sample was then centrifuged at 4 °C and 10,000 rpm for 90 minutes for the density separation. The upper phase was then filtered through a polycarbonate filter membrane with 0.2 µm pores (obtained from Legionella Kit from rqmicro AG, Switzerland) to retain bacterial cells, which were then resuspended in 16.5 mL minimal medium without additional carbon source by through shaking of the filter membrane in the medium by a vortexer. 1 mL of this suspension was added to 4 mL of minimal medium supplemented with 1.4 mg of dPLA and incubated at room temperature of 21 °C for two weeks.

Scanning electron microscopy (SEM) of dPLA particles

Deuterated PLA was suspended in MilliQ water by exposing the sample to an ultrasonic bath for 1 min. A 5 µL droplet of the dPLA suspension was applied on a Si wafer and freeze-dried. All SEM images were measured on a Sigma 300 VP Field Emission SEM (FE-SEM) from Carl Zeiss AG, Germany, equipped with a secondary electron detector. An aperture of 30 µm was chosen for all

samples. Due to the large particle size heterogeneity, it was not possible to measure all particles with the same settings. Therefore, large dPLA MP particles were measured under 500 x magnification with the secondary electron detector with a voltage of 1 kV (see Figure S8 for 4 out of the 9 analyzed SEM images). Small particles were sputtered twice with platinum for 90 s with 15 mA and 10 kV and measured under 10.000 x magnification with the same detector with a larger voltage of 5 kV(see Figure S9 for 6 out of the 22 analyzed SEM images). Due to the shadows that typically appear in images obtained with a secondary electron detector, the images could not be automatically processed. Instead, the open-access software ImageJ[2] was used to roughly estimate particle sizes manually. The sizes of 115 particles under 500x magnification and 231 particles under 10.000x magnification are summarized in the particle size distributions in Figure S10, and the SEM images with bars of the measured particles are shown in Figures S8 & S9.

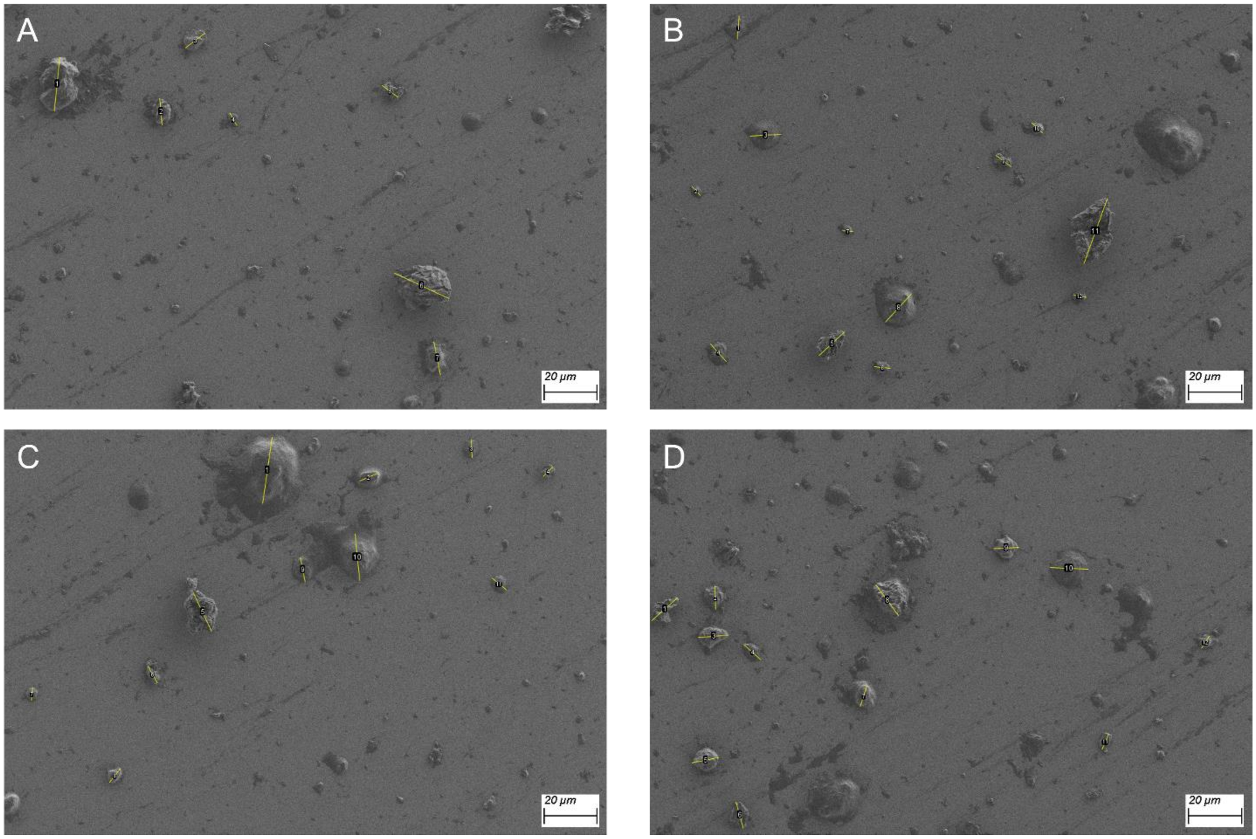

Figure S 13 Scanning electron microscopy images of a dPLA suspension in MilliQ water. The images were obtained with 500x magnification, a voltage of 1 kV, and an aperture of 30 µm with a secondary electron microscope. The scale bars in the lower right corner of the images correspond to 20 µm. The particles which were measured for the size distribution are marked with a yellow line.

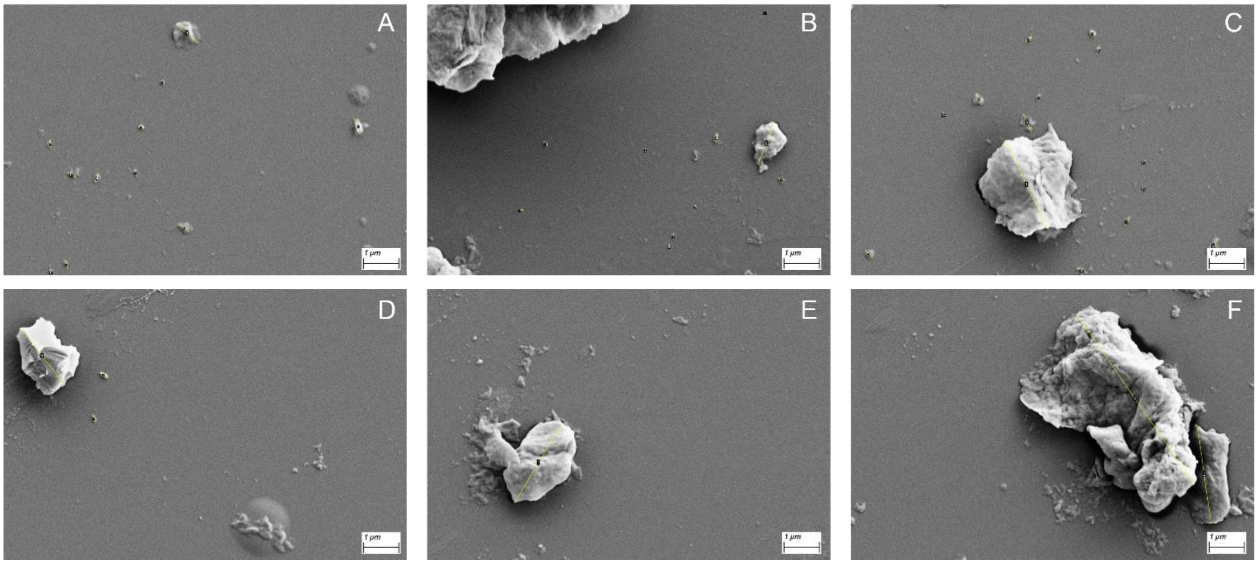

Figure S 14 Scanning electron microscopy images of a dPLA suspension in MilliQ water. The sample was sputtered with Pt to increase the conductivity. The images were obtained with 10.000x magnification, a voltage of 5 kV, and an aperture of 30 µm with a secondary electron microscope. The scale bars in the lower right corner of the images correspond to 1 µm. The particles which were measured for the size distribution are marked with a yellow line.

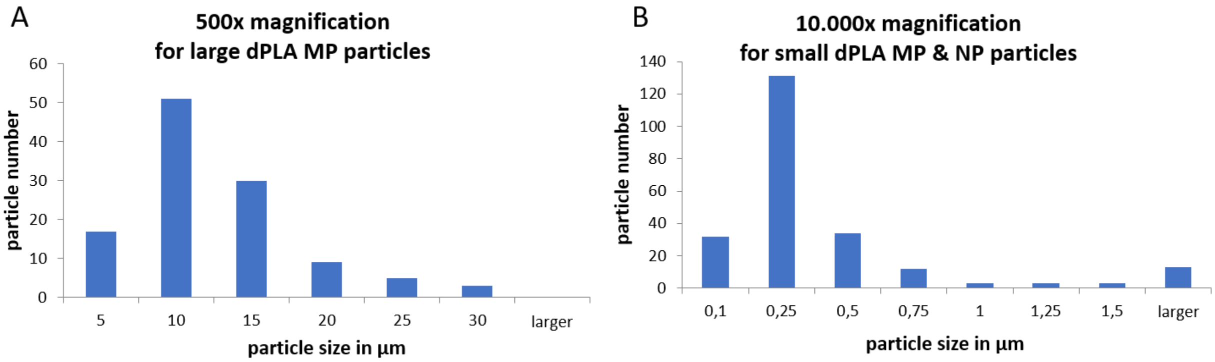

Figure S 15 Deuterated PLA particles size distributions were roughly estimated from the SEM images in Figures S8 & S9. A. 115 large particles were measured under 500x magnification. B. 231 sputtered, smaller particles were measured under 10.000x magnification.

Size-exclusion Chromatography (SEC) of dPLA

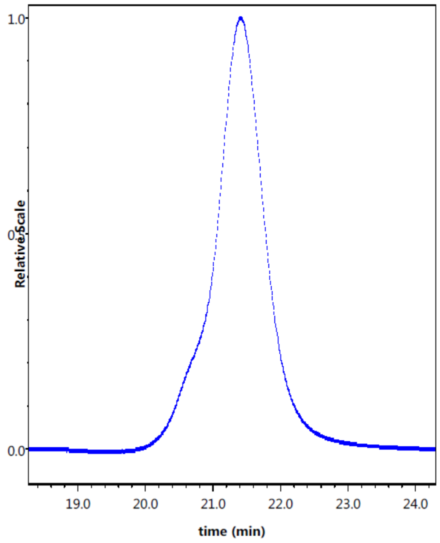

Figure S 16 SEC RI-trace of dPLA. The number average molecular weight (Mn) of the dPLA sample is Mn = 3900 g/mol with narrow molecular weight distribution (Mw/Mn = 1.16, with Mw being the weight average molecular weight).

Data processing

The data for each figure was prepared with a different inhouse written Python script. The data for each mean spectrum is stored in a separate folder. The script allows to automatically import one file after another and applies the same pretreatment to all graphs within one figure.

Mean spectra:

- The Si band in all attached daily silicon wafer reference spectra is fit by a Gaussian function and the daily wavenumber shift is calculated as difference of the peak position from the intentended Raman band at 520 cm^{-1} and stored in a dictonary.
- Wavenumbers and intensities of the single cell spectra are imported from all datafiles within one folder. And the following code is performed per single spectrum:
  - The wavenumber is corrected with the Si wafer reference dictonary based on the date included in name of the spectrum.
  - Spectra are cropped to the region of interest (705 – 3195 cm^{-1}).
  - Spectra are smoothed based on a Savitzky-Golay filter (window length: 7, polynominal order: 3).
  - The baseline is corrected with the rubberband function from the open source RamPy baseline package.
  - The normalization depends on the figure:
    - Fig. 3A+5A: Integral of the C-D Raman band plus the C-H Raman band equals 1.
    - Fig. 3B: Integral of the C-D vibrations Raman band equals 1.
    - Fig. 6A: Min-max scaling: min = 0, max = 1.
- The intensities of all spectra of one category (e.g. all spectra of the D_2O reference) are then summed up per each wavenumber and divided by the number of single spectra to achieve the mean spectrum of the population.
- The plots are then created using the following open-source libraries: Matplotlib, Seaborn and Pandas.

Quantitative analysis – C-D and C-H Raman bands:

- Gaussian profiles were fit to specific C-D and C-H Raman bands. Here, five Gaussian functions were chosen instead of the before mentioned three Gaussian fits, assigned to lipids, proteins, and DNA. The model of the three Gaussian functions did not fit well with the C-D signature of spectra of *S. koreensis* grown on dPLA due to the additional Raman band of strongly deuterated lipids. A model with five Gaussians turned out to describe the C-D signature with the highest accuracy and was therefore chosen for all C-D and C-H bands. The integrals of the Gaussian functions were calculated and stored to a text file.
  - As a fitting function, five Gaussians were summed up.
  - The peak fitting was performed with the SciPy package optimize.curve_fit.
  - To make the peak fitting more reproducible, certain boundary conditions were chosen:

- **C-D Raman bands** (1950 cm^{-1} – 2400 cm^{-1}):
  amplitude: 0 – 0.01 a.u., initial guesses differ for five Gaussians: 0.00006, 0.00008, 0.00006, 0.00004, and 0.00004 a.u.
  band width: 1 – 50 cm^{-1}, initial guess: 15 cm^{-1}
  intensity offset (y_0): 0 – 0.001, initial guess: 0

	Lower limit	Upper limit	Initial guess
Gaussian 1	2060 cm^{-1}	2080 cm^{-1}	2070 cm^{-1}
Gaussian 2	2103 cm^{-1}	2123 cm^{-1}	2113 cm^{-1}
Gaussian 3	2138 cm^{-1}	2158 cm^{-1}	2148 cm^{-1}
Gaussian 4	2186 cm^{-1}	2206 cm^{-1}	2196 cm^{-1}
Gaussian 5	2213 cm^{-1}	2233 cm^{-1}	2223 cm^{-1}

- **C-H Raman bands** (2750 cm^{-1} – 3030 cm^{-1}):
  amplitude: 0 – 0.01 a.u., initial guesses differ for five Gaussians: 0.0009, 0.005, 0.005, 0.005, and 0.003 a.u.
  band width: 1 – 50 cm^{-1}, initial guess: 15 cm^{-1}
  intensity offset (y_0): 0 – 0.001, initial guess: 0

	Lower limit	Upper limit	Initial guess
Gaussian 1	2840 cm^{-1}	2860 cm^{-1}	2848 cm^{-1}
Gaussian 2	2861 cm^{-1}	2890 cm^{-1}	2880 cm^{-1}
Gaussian 3	2895 cm^{-1}	2920 cm^{-1}	2910 cm^{-1}
Gaussian 4	2921 cm^{-1}	2938 cm^{-1}	2932 cm^{-1}
Gaussian 5	2955 cm^{-1}	2980 cm^{-1}	2970 cm^{-1}

  - The integrals were calculated based on the optimized Gaussian functions from the curve fit with a trapezoidal function provided in the SciPy integrate package. However, the offset intensity y_0 was neglected since it only arises from the baseline.
  - All data for the C-D vibrations of one category (e.g. *S. koreensis* incubated with dPLA for 3 weeks) was stored with the according filenames of the single cell spectra to a .txt file.
  - Once everything was calculated for the C-D vibrations, the same was performed for the C-H vibrations.
- The files for all categories, including the data of the different peak areas, were then used by a second code, read-in, and the according graphics, including boxplots and strip plots were made.
  - Strip plots and boxplots were created using the following open-source libraries: Matplotlib, Seaborn and Pandas.

Quantitative analysis – Resonance Raman spectra:

- Spectra were cropped to the region of 700 cm^{-1} – 2000 cm^{-1} and a wavenumber correction was performed as previously described for the full biomass spectra.
- Due to the high signal intensities, no smoothing and baseline correction was required.
- The spectra were normalized to the minimal and maximum intensities.
- Mean spectra were gained by summing all spectra of one category and dividing by the number of spectra.
- To determine the red-shift of the ν_1 Raman band, it was fit by a single Gaussian profile with the SciPy package optimize.curve_fit. In case of the presence of two Raman bands, caused by isotopologues, the most intense Raman band was fitted by choosing according boundary conditions for the fit.
    - Initial guesses and fitting boundaries:
      Amplitude: 0.5 – 1, initial guess: 1 (due to min-max scaling)
      Peak position (depending on category to find the most intense Raman band): 1500 cm^{-1} - 1524 cm^{-1}, 1474 cm^{-1} - 1504 cm^{-1}, 1474 cm^{-1} - 1496 cm^{-1}, or 1474 cm^{-1} - 1487 cm^{-1}, initial guesses: 1510 cm^{-1}, 1490 cm^{-1}, and 1476 cm^{-1}
      Band width: 0 – 17 cm^{-1}, initial guess: 10 cm^{-1}
      Intensity offset (y_0): 0 – 0.4 a.u., initial guess: 0 a.u.
    - The fits were then displayed in a simple plot to check for correctness, especially for the Raman spectra of the samples, where isotopologues were formed.
- The ν_1 Raman band positions were then plotted as categorial swarmplots with the according seaborn package.

Extended discussion on different plausible biosynthesis pathways of amino acids and fatty acids with either lactate or glucose as substrate

Detailed descriptions of all mentioned metabolic pathways can be looked up in the book ‘Stryer Biochemie’[3], which is also available in an international edition.

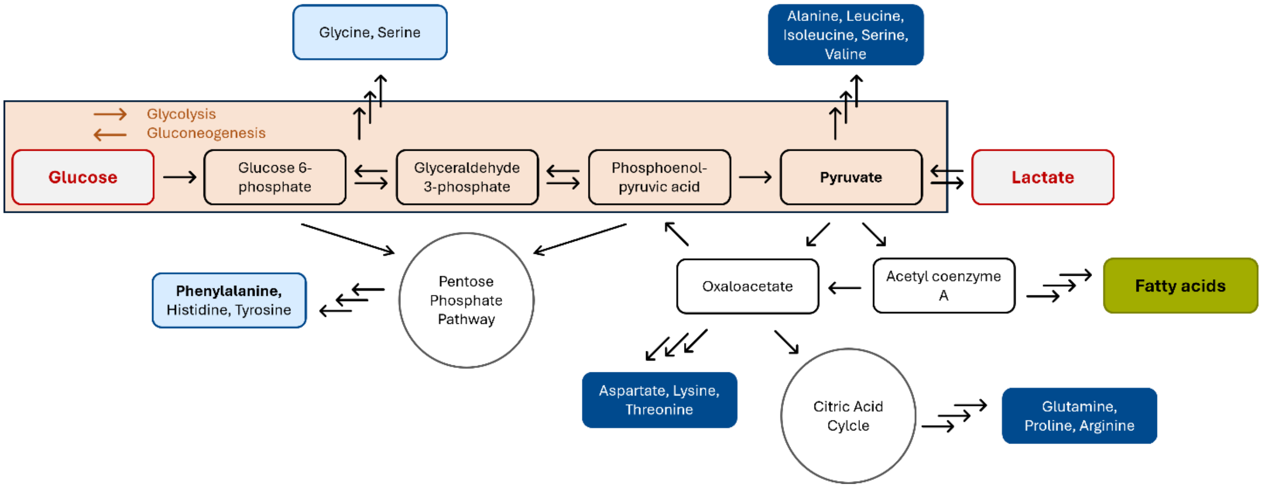

Figure S 17 Metabolic pathways from glucose and lactate to fatty acids (green box) and amino acids (light and dark blue boxes). Glucose undergoes glycolysis and serves as a precursor for the synthesis of all the depicted amino acids and fatty acids. In contrast, the conversion of lactate into pyruvate indicates a preferential pathway for the synthesis of fatty acids and the amino acids shown in the dark blue boxes. To produce other amino acids (light blue boxes), such as phenylalanine, pyruvate must first enter the gluconeogenesis pathway, which is an energy-intensive process.

Lactate as substrate

The lactate dehydrogenase can directly convert lactate into pyruvate[4], which can then be transformed into acetyl-CoA, the building block for almost all fatty acids (see Figure S17). During the fatty acid synthesis, NADPH acts as a reducing agent and provides additional hydrogen besides the hydrogen already present in acetyl-CoA. The NADPH hydrogen can hypothetically also be derived from lactate via gluconeogenesis of pyruvate to glucose-6-phosphate and the subsequent pentose-phosphate-pathway. Especially due to the direct pyruvate formation, lactate could be expected to provide many hydrogen (or in our case deuterium) atoms to the final lipids.

Opposing this straight-forward biosynthesis of lipids, proteins and DNA require a broader set of precursors, which are used for biosynthesis of different amino acids. The most important precursors for the carbon backbone of amino acids are oxalacetate, pyruvate, ribose-5-phosphate, phosphoenolpyruvate, erythrose-4-phosphate, α-ketoglutarat and 3-phosphoglycerate, which are intermediates of the glycolysis, pentose-phosphate-pathway and citrate cycle. Due to the length of the biosynthesis routes, it is more likely that deuterium from the initial lactate is exchanged with hydrogen from water or reducing agents.

In conclusion, metabolic pathways of deuterated lactate might lead to stronger deuteration of lipids than of other biomolecules.

Glucose as substrate

During glycolysis, one glucose molecule can be transformed into two pyruvate molecules. This pyruvate could be either oxidized to CO_2 to produce energy in form of ATP or it could be used for other metabolic pathways, such as fatty acid synthesis, as described above (see Figure S17). However, during 9 enzymatically catalyzed steps of glycolysis, the intermediates can already be introduced into the other metabolic pathways to produce a broad variety of biomolecules. Therefore, glucose appears to be a more versatile precursor for different biomolecules and the deuterium label is expected to be more evenly distributed.

Estimation of deuterium content in the biodegradation experiments

Assumptions:

i) The entire deuterium of the labelled dPLA would exchange with hydrogen atoms of the aqueous medium.
ii) The aqueous medium is estimated to consist of 100 % H_2O.

Given numbers:

V(medium) = V(H_2O) = 10 mL → m(H_2O) = 10 g

M(H_2O) = 18 g/mol

m(dPLA) = 3 mg

M(dPLA unit) = 76 g/mol

Calculations:

n = m / M

n(H_2O) = 0,556 mol → n(H) = 2 · n(H_2O) = 1,112 mol = 1112 mmol

n(dPLA unit) = 0,039 mmol → n(D) = 4 · n(dPLA unit) = 0,156 mmol

ratio D / (H+D) = 0,014 %

Interpretation:

If all deuterium of dPLA would end up in the aqueous medium, it would only account for 0,014% of all hydrogen atoms. Therefore, the deuteration would be so low, that it would not be detectable with Raman microspectroscopy.

An extended explanation of apparent isotope shifts of carotenoid Raman bands upon deuteration

Depending on the way vibrations are coupled, the relative Raman bands can be either red- or blue-shifted. If the carotenoid C-C stretching vibrations are coupled to C-H bendings, they are red-shifted and appear at 1156 cm-1. Without coupling, they would appear at higher wavenumbers. Now, if the molecule is deuterated, the C-D bendings appear at lower wavenumbers (<1000 cm^{-1}) due to the higher mass of deuterium. They thereby get decoupled from the C-C stretching vibrations, which then have a band at their originally uncoupled position, which is blue-shifted compared to their previous Raman band.

References

1. Wang, Y., et al., *Raman–deuterium isotope probing to study metabolic activities of single bacterial cells in human intestinal microbiota.* Microbial Biotechnology, 2020. **13**(2): p. 572-583.
2. Schneider, C.A., W.S. Rasband, and K.W. Eliceiri, *NIH Image to ImageJ: 25 years of image analysis.* Nature Methods, 2012. **9**(7): p. 671-675.
3. Jeremy M. Berg, J.L.T., Gregory J. Gatto jr., Lubert Stryer, *Stryer Biochemie*. 2017, Springer Spektrum Berlin: Heidelberg, Germany. p. 1401.
4. Feldman-Salit, A., et al., *Regulation of the Activity of Lactate Dehydrogenases from Four Lactic Acid Bacteria *.* Journal of Biological Chemistry, 2013. **288**(29): p. 21295-21306.
